# The role of tumor microenvironment on cancer stem cell fate in solid tumors

**DOI:** 10.1186/s12964-023-01129-w

**Published:** 2023-06-16

**Authors:** Sara Razi, Amin Haghparast, Sepide Chodari Khameneh, Amin Ebrahimi Sadrabadi, Fatemeh Aziziyan, Maryam Bakhtiyari, Mohsen Nabi-Afjadi, Vahideh Tarhriz, Arsalan Jalili, Hamidreza Zalpoor

**Affiliations:** 1 Vira Pioneers of Modern Science (VIPOMS), Tehran, Iran; 2 GenomeFan, Tehran, Iran; 3 Department of Stem Cells and Developmental Biology, Cell Science Research Center, Royan Institute for Stem Cell Biology and Technology, ACER, Tehran, Iran; 4 Cytotech and Bioinformatics Research Group, Tehran, Iran; 5 Department of Biochemistry, Faculty of Biological Sciences, Tarbiat Modares University, Tehran, Iran; 6 Network of Immunity in Infection, Malignancy & Autoimmunity (NIIMA), Universal Scientific Education & Research Network (USERN), Tehran, Iran; 7 Department of Medical Laboratory Sciences, Faculty of Allied Medicine, Qazvin University of Medical Sciences, Qazvin, Iran; 8 Infectious and Tropical Diseases Research Center, Tabriz University of Medical Sciences, P.O. Box 5163639888, Tabriz, Iran; 9 Parvaz Research Ideas Supporter Institute, Tehran, Iran; 10 Shiraz Neuroscience Research Center, Shiraz University of Medical Sciences, Shiraz, Iran

**Keywords:** Cancer stem cells, Tumor microenvironment, Metabolism, Solid tumors, EMT, Cellular plasticity, Signaling pathways

## Abstract

In the last few decades, the role of cancer stem cells in initiating tumors, metastasis, invasion, and resistance to therapies has been recognized as a potential target for tumor therapy. Understanding the mechanisms by which CSCs contribute to cancer progression can help to provide novel therapeutic approaches against solid tumors. In this line, the effects of mechanical forces on CSCs such as epithelial-mesenchymal transition, cellular plasticity, etc., the metabolism pathways of CSCs, players of the tumor microenvironment, and their influence on the regulating of CSCs can lead to cancer progression. This review focused on some of these mechanisms of CSCs, paving the way for a better understanding of their regulatory mechanisms and developing platforms for targeted therapies. While progress has been made in research, more studies will be required in the future to explore more aspects of how CSCs contribute to cancer progression.

Video Abstract

**Supplementary Information:**

The online version contains supplementary material available at 10.1186/s12964-023-01129-w.

## Introduction

Cancer is still one of the primary reasons of death all around the world with several complications such as metastasis, heterogeneity in cells, invasion, relapse, and therapy resistance [1]. In the last two decades, the discovery of the origin of cancer has led to a better understanding of the mechanism of malignancies. In this line, the model of cancer stem cells (CSCs) has been widely accepted in different malignancies as potential factors in charge of invasion and therapy resistance [2–5]. Despite advances in screening programs and the development of new immunotherapy methods, eradication or representation of a defined method to identify, recognize, and isolate the population of turmeric cells has not yet succeeded and remains unknown. One of the challenges ahead of the eradication of CSCs is reckoned to stem from the plasticity and heterogeneity that these cells show in the microenvironment of a tumor [6]. This plasticity could take root in the metabolism of these cells, which has been introduced to be one of the ways through which this fraction of cells could support their feature of growth and tumorigenesis [7]. The metabolism of CSCs has received special attention as the key to adjusting to the severe condition of the tumor microenvironment (TME), which contributes to cancer cells thriving, expanding, and overcoming immune cells [8]. The plasticity and heterogeneity of CSCs in their metabolism pathways could be rooted in different factors. One of which is assumed to be the TME consisting of factors like tumor-associated macrophages (TAM), cancer-associated fibroblasts (CAF), endothelial cells (ECs), immune cells and the received signals from the presented cells in TME, playing a vital role in influencing and regulating the population of CSCs and their metabolism reprogramming [8, 9]. Therefore, considering the significance of TME in the regulation of the metabolism of CSCs, in this review, we will focus on the effects of mechanical forces on CSCs and the metabolism of CSCs, players of the TME and the influence of the TME on regulating the metabolism of CSCs.

### The origin of cancer stem cells and their contribution to cancer progression

Tumor-initiating cells (TICs) also known as CSCs are subpopulations of tumor cells that initiate tumors and cause relapses. Cells that can self-renew are designated as cancer stem cells. These cells divide and give rise to other cells that give rise to different kinds of cancer [10–12]. CSCs develop from tumor progenitor cells, stem cells, or dedifferentiated cells that acquire CSC characteristics during tumor initiation. Transformation can occur during regeneration and as a result of infections, toxins, radiation, or metabolic influences causing mutations [13]. The transformation can also occur as a result of infections, toxins, radiation, or metabolic influences. At this time, tumor suppressors are inactivated promoting uncontrolled growth of the cells [14]. Consequently, stem cells acquire stem cell characteristics as a result of de-differentiation. For stem cells to transform, different genomic changes are needed that allow them to proliferate in uncontrolled, niche-independent ways [15]. It is believed that stem cells and their progeny can be transformed by only a few genomic changes since stem cells have unlimited growth potential. Even differentiated intestinal epithelial cells can become CSCs in mice, according to recent studies [16, 17]. The liver has also been shown to produce tumors from adult differentiated cells, tissue-resident stem cells, or their progeny [18, 19]. Only the CSC population can initiate tumor growth in tumors generated from CSCs, resulting in a unidirectional hierarchy. To maintain their pool of CSCs, CSCs divide asymmetrically at tumor initiation. Transient amplifying cells are generated from asymmetric divisions and undergo symmetric divisions, resulting in high proliferation rates [20, 21]. For the first time, the hypothesis of CSCs was proved by Lapidot et al. [22], who found out that a rare population isolated from myeloid leukemia (AML) can initiate tumorigenesis in severe combined immune-deficient (SCID) mice [22]. This minority subgroup whose footsteps have been found in numerous tumors, including melanoma [23], breast cancer [24], AML [25], gastrointestinal cancer [26], colorectal cancer [27], glioblastoma [28], pancreas cancer [29], and lung cancer [30] is believed to derive from normal tissue stem cells or the dedifferentiation of normal cancer cells. Resistance to therapy, recurrence, tumor growth, and metastasis have been attributed to the presence of this small fraction of the cells in their residence tumor [31, 32]. Properties like self-renewal, remaining in the G0 phase, and expression of molecules related to the drug efflux transport system in CSCs contribute to drug resistance, and the capacity to initiate tumorigenesis [28, 33]. Concerning the fact that the markers expressed by CSCs are also expressed by the other populations of stem cells like adult tissue residents and embryonic stem cells that can also vary among different tumor types lead to limiting the specifying biomarkers of CSCs [34]. However, over-expression of surface markers such as CD133, CD44, epithelial cell adhesion molecule (EpCAM, CD326) [35–42], homeobox protein NANOG, octamer-binding transcription factor 4 (OCT4), sex-determining region Y HMG-box 2 (SOX2) [43] sphere formation [44], aldehyde dehydrogenases 1A1 (ALDH1A1) activity [45] and ATP-binding cassette sub-family G member 2 (ABCG2) [46] have been detected and used for isolation of CSCs. It is not surprising that CSCs take advantage of signaling pathways like Wnt/β-catenin, c-MYC, Janus kinase /signal transducer and activator of transcription (JAK/STAT), Hedgehog/Notch, etc. [47, 48] to preserve some of their properties like mechanical forces and metabolism shifting and regulation, plasticity, self-renewal, etc. [49]. It is notable that metabolism alterations in these cells are closely related to the mentioned signaling pathways, which will be discussed in the next parts.

### Metabolism feature of CSCs

**Fig. 1:**
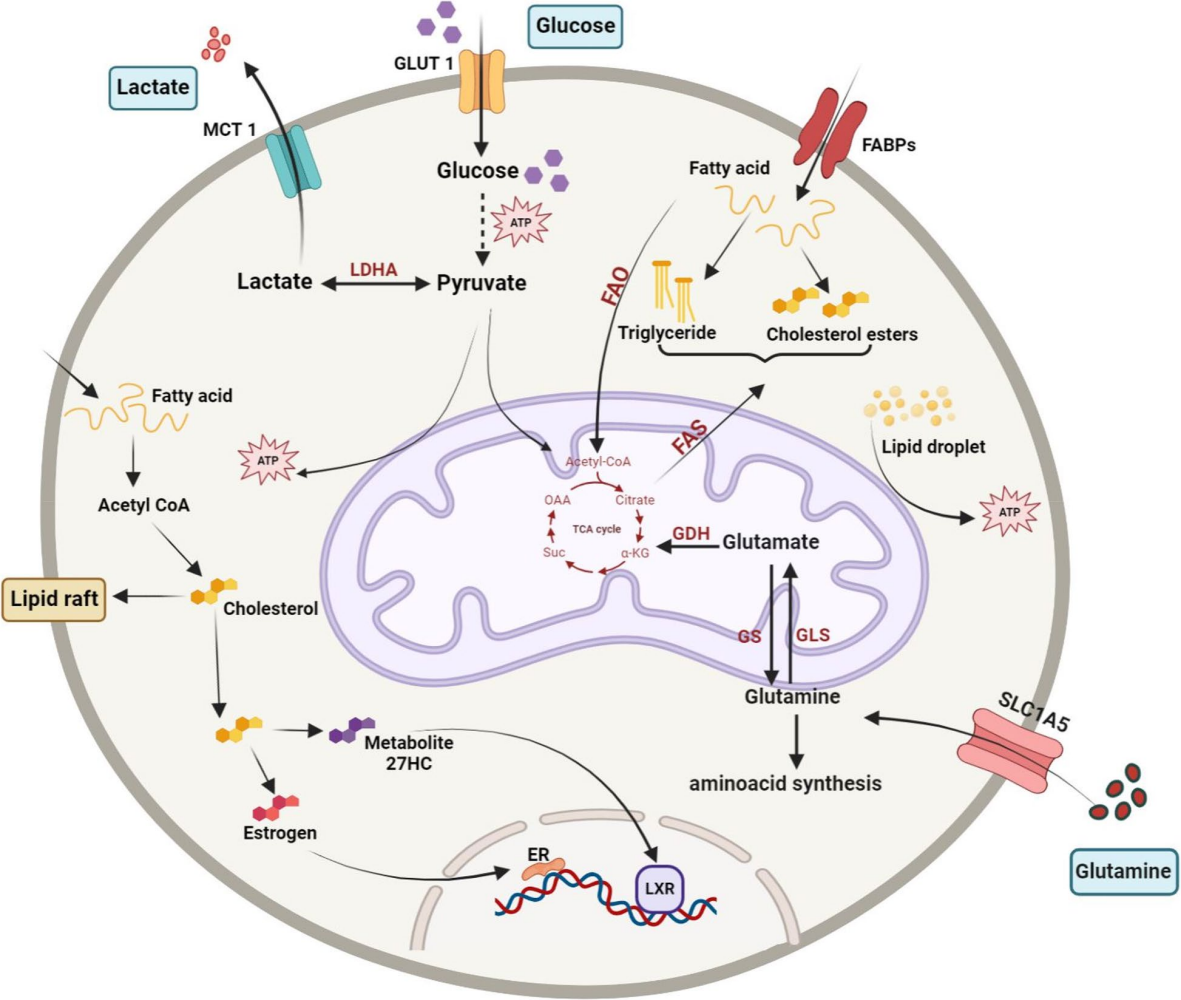
Metabolic adaptation in CSCs and cancer cells. Even when there is a sufficient supply of oxygen, cancer cells frequently adopt the Warburg effect or aerobic glycolysis, relying on glycolysis rather than OXPHOS for ATP production. As a result, the pyruvate is turned into lactate and transferred outside of the cell, where it acidifies the tumor microenvironment and creates an immune-suppressive environment. Additionally, glutamine becomes more important to cancer cells for anabolic processes (such as the production of nucleotides and other amino acids) that promote cell growth and replenish the TCA cycle. In addition, glutamine plays a crucial role in glutathione production, which is essential for chemo-resistance. In cancer, fatty acid oxidation (FAO) and fatty acid synthesis (FAS) are increased to supplement glycolysis for energy and provide the necessary membrane components for accelerated cell development. Furthermore, the composition of their membranes and the signaling that promotes proliferation and invasion in cancer cells rely on the production of cholesterol

To survive in the wide range of micro-environmental conditions they experience, CSCs are likely designed to obtain energy from various sources, depending on the available substrates. There is sufficient support for a glucose-based and oxidative-based metabolism [50]. Furthermore, CSCs may use different amino acids as fuel, such as glutamine and lysine. Acetyl-CoA is generated from glycolysis, and fatty acid oxidation is catabolized by the tricarboxylic acid cycle (TCA) cycle and oxidative phosphorylation (OXPHOS) in normal cells' mitochondria to make ATP. Through the well-known Warburg effect, cancer cells, unlike normal cells, increase the glycolytic flux in aerobic conditions [50]. Additionally, the glycolytic degradation of glucose creates the building blocks for the biosynthesis of nucleotides and amino acids. As a result, the transition from oxidative to glycolytic metabolism effectively gives cancer cells the ability to endure difficult conditions with low oxygen levels. It causes cancer cells to proliferate, move to distant regions, and attack other tissues. It has been established that CSCs have a different metabolism from non-CSCs, whose phenotype, at least in part, resembles that of regular stem cells, which mainly consume glucose. The development of pluripotent markers coincides with metabolic reprogramming toward glycolysis and indications of mitochondrial involution in induced pluripotent stem cells [51]. However, many studies have suggested that secondary pathways such as fatty acid oxidation, PPP pathway, and glutaminolysis, as well as mitochondria and OXPHOS, may be essential for CSC metabolism [52] (Fig. 1). The uncontrolled expansion of tumor cells requires to be supported by increased uptake of nutrients [53, 54]. Metabolism adaptation has been introduced as one of the ways through which cancer cells get to supply their energy demands. The difference between normal cell metabolism and cancer cells metabolism such as increased glucose uptake and lactate production in the presence of oxygen was first noticed by Otto Warburg [55, 56]. The metabolism shifting can be applied to CSCs, although in recent experiments, the metabolism of CSCs has been the topic of debate, as controversial data on whether CSCs mostly rely on glycolysis or mitochondria-related metabolism have been reported [57].

#### Glucose is a source of energy for CSCs

The significance of glucose for CSC preservation and proliferation in various cancer cells, including the brain, lung, breast, liver, osteosarcoma, nasopharyngeal cancer, and glioblastoma, has been thoroughly demonstrated by numerous studies. Liu et al. recently showed that a subpopulation of cells with stem-like characteristics primarily relies on glucose as a fuel source by employing a panel of cancer cell lines. In addition, glucose was able to raise the proportion of cancer stem-like cells, which had increased levels of various glycolytic enzymes and lactate generation. The number of CSCs was also decreased, and their capacity to develop tumors in vivo was interfered with by inhibiting glycolysis [58–62]. When the activity of the mitochondrial complex I was blocked for the loss of FBP1, an acceleration of glycolysis and the acquisition of stem characteristics were seen. Additionally, it has been demonstrated that overexpressing FBP1 reduces the number of cancer cells with stem-like features in basal-like breast cancer cells and prevents the formation of tumor spheroids in vivo. FBP1 promotes the gluconeogenic pathway while suppressing glycolysis [61, 63]. Further supporting these findings, Shen et al. recently showed that compared to CD133-cells, a subgroup of hepatic cancer cells selectively activate aerobic glycolysis and inhibit the gluconeogenic pathway. The glycolysis rate and capacity of the CSC-like subgroup were increased, and the glycolytic enzymes HK2, GLUT1, pyruvate dehydrogenase kinase (PDK), and PGAM1 were all upregulated. In contrast, the gluconeogenic enzymes PEPCK and G6PC were down-regulated [60]. These findings imply that the primary catabolic pathway of CSCs from various tumor types is glycolysis, which inhibits anabolic de novo synthesis. This has also been mentioned in colorectal cancer (CRC), where CSCs have recently been found to have an odd metabolic signature. They combined high-resolution unbiased metabolomics with transcriptome analysis of five microarray datasets of CD133^+^ and CD133 cell subpopulations derived from CRC cell lines and patients. This made it possible to depict the metabolic activity of CSCs, which was characterized by up-regulated fatty acid production and down-regulated expression of genes and metabolites from the glycolytic pathway and TCA cycle [64]. Recently, the metabolic profile of breast cancer cells grown as spheroids or in adherent conditions was examined using high-throughput data from proteome and targeted metabolomics analyses. The enhanced activity of the pyruvate kinase M2 isoform, lactate dehydrogenase, and glucose 6-phosphate dehydrogenase in cancer stem-like cells suggests a switch from mitochondrial oxidative phosphorylation toward fermentative glycolysis. In Goidts et al., RNA interference (RNAi) was used to screen the entire human kinome and phosphatome to identify genes and pathways essential for glioblastoma CSC survival. They discovered numerous genes involved in metabolism, particularly the glycolytic enzymes including 6-phosphofructo-2-kinase/fructose-2,6-biphosphatase 4 (PFKFB4), pyruvate kinase M2 hypotype (PKM2), and PDK-1, which were crucial for maintaining brain CSCs [65, 66]. Together, these findings support the unique function of glucose as the primary fuel for CSCs, and oxidative pathways may be adversely affected by the low oxygen availability in the hypoxic CSC niche. In this context, it has been demonstrated that TICs isolated from human glioblastoma xenografts use glycolysis to produce ATP and prefer low-oxygen environments to maintain their stemness characteristics and tumor-forming potential [67]. Hypoxia's role in CSC proliferation has been thoroughly studied, and it has been linked to glucose dependence, especially in the quiescent phenotype of CSCs. Mahase and colleagues have explored the potential for various processes, such as CSC proliferation and metabolic changes, to explain the resistance to antiangiogenic medications in the therapeutic management of glioblastoma patients. It has been demonstrated that the formation of intra-tumor hypoxia following the injection of antiangiogenic drugs increases the subpopulation of cells with stem characteristics in glioblastoma, breast cancer, and lung cancer [68–70]. Hypoxia-inducible factor-1 (HIF-1) enables the transcription of genes involved in glucose regulation and ATP synthesis and has been related to an increase in the ALDH^+^ population. Hexokinase-2, the first enzyme in the Embden-Meyerhoff/glycolytic pathway, is abnormally expressed by glioma cells in perinecrotic regions. Its overexpression is related to glioblastoma cell proliferation and aerobic glycolysis. Additionally, an increase in PDK-1 caused by HIF-1α-mediated action prevents pyruvate dehydrogenase activity and TCA cycle entry [69–71]. Glycolysis, which involves the production of nicotinamide adenine dinucleotide (NADH), pyruvate, and two ATP molecules, seems to be the preferred metabolism pathway by cancer cells. Cancer cells change their metabolism pathways to glycolysis to maintain abnormal growth, while normal cells are more dependent on OXPHOS, ATP, lactate, and pyruvate production [72]. The reprogrammed metabolism to glycolysis has been shown to take over in different types of CSCs like breast cancer, osteosarcoma [73], ovarian CSCs [74], lung and colorectal CSCs [58], hepatocellular carcinoma [75], brain cancer [76]. Similar evidence for metabolism switching to glycolysis has been observed in induced pluripotent stem cells (iPS) [77]. Glycolysis could guarantee fast proliferation through glucose-6-phosphate (G6P) production that can be used in the formation of ribose groups and, consequently, the synthesis of nucleotides that is necessary for the rapid replication of cancer cells [78–80]. Increased expression of genes imprinted in glucose-related metabolism genes like glucose transporter 1 (GLUT-1), PDK-1, hexokinase 1 (HK-1), and c-Myc makes up the expansion of the population of CSCs, extends the lifespan of cells and their immortalization [77, 81]. Furthermore, the interference of oncogene transcription factors, including NANOG, OCT4, Wnt, Myc, K-Ras, and HIF-1α in the shift of CSCs to glycolysis, could provide beneficial support [82–85], which is consistent with the fact that reduction of mitochondrial-related metabolism and downregulation mitochondrial genes is associated with enhanced expression of epithelial-mesenchymal transition (EMT) genes linked to stemness [86]. Furthermore, maintaining stemness in some cancer types seems to be closely related to reduced mitochondrial-dependent metabolism [73, 87, 88]. On the other hand, considering the enhanced mitochondrial DNA and mature cell gene expression and decreased expression of stemness-related genes during differentiation, indicate that CSCs would likely tend to shift glycolysis [89]. Above all, low reactive oxygen species (ROS) production, enhanced detoxification system, creation of an acidic environment, and escaping the immune system cells, which consequently help the invasion and metastasis, are other beneficial effects for CSCs through metabolic reprogramming [90–92].

#### The OXPHOS phenotype in CSCs

Contrary to the conventional belief that CSCs primarily exhibit a glycolytic phenotype, several studies indicate that CSCs preferentially utilize mitochondrial respiration and oxidative metabolism. Numerous independent researchers have presented evidence of decreased glycolytic flow and enhanced mitochondrial-driven ATP generation. For example, it has been discovered that mitochondrial OXPHOS and fatty acid oxidation enzymes are up-regulated in CSCs isolated from individuals with ovarian cancer [93]. Consequently, current analysis and comparisons of the metabolic characteristics of spheroids developed from both ovarian and cervical carcinoma cells were completed. Intriguingly, the researchers noted that spheroid CSCs had a TCA cycle metabolism that is different from non-CSCs. Gao et al. recently FACS sorted CSCs from small cell lung cancer cells using a similar experimental strategy to examine their metabolic condition. Compared to their non-stem counterpart, CSCs were discovered to have a stronger dependence on OXPHOS and mitochondrial activity [94, 95]. It has been shown that CSCs isolated from gliomas use less glucose, generates less lactate, and keep their levels of ATP from oxidative phosphorylation high. In CD133^+^ human glioblastoma cells, there has been a similar tendency toward using mitochondrial respiration over glycolysis, with a mechanism relying on the insulin-like growth factor 2 mRNA-binding protein. The researchers specifically showed that IMP2, which is involved in controlling oxygen consumption rate (OCR), mitochondrial mass, and the expression of various stemness markers, including CD133, SOX2, OCT4, and NANOG, is more highly expressed in CD133^+^ glioblastoma cells. Given that IMP2 directly interacts with numerous genes for mitochondrial complexes to drive the assembly of complexes I and IV, it is reasonable to suppose that the increased IMP2 expression found in glioblastoma CSCs may result from the cells' high need for OXPHOS [96, 97]. In a mouse model of pancreatic cancer, Viale et al. also examined the population of dormant cells that survived the deletion of the oncogene RAS. It has been established that these inactive cells have stem-like characteristics and depend on mitochondrial activity and oxidative phosphorylation rather than glycolysis and glutaminolysis [98]. Notably, the transcription co-activator peroxisome proliferator-activated receptor gamma, co-activator 1 alpha (PPARGC1A, referred to as PGC-1), has been linked to the ability of cancer cells to metastasize. As demonstrated by employing human invasive breast tumor samples, PGC-1 has been clinically shown to relate oxygen consumption, OXPHOS, and mitochondrial biogenesis with the increased migratory and invasive potential of cancer cells [99]. The upregulation of PGC1 has been seen in circulating tumor cells and breast CSCs, where its suppression decreases stemness qualities, supporting the involvement of PGC1 in CSC maintenance and proliferation via mitochondrial activity. These findings suggest that the biology of CSCs depends on intact mitochondrial activity and function. Since CSCs produce more mitochondrial mass and membrane potential, more mitochondria-derived ROS are produced, and they consume more oxygen than differentiated cells in the tumor bulk; mitochondrial biogenesis is recognized as a crucial characteristic of CSCs in this setting [93, 100–106]. According to a recent study, brain tumor-initiating cells, which show increased mitochondrial fission mediated by dynamin-related protein 1, play an essential role in mitochondrial dynamics (DRP1). It's remarkable to note that DRP1, which inhibits mitochondrial fission by severing the membrane stalk between two developing daughter mitochondria, was associated with a poor prognosis in glioblastoma, indicating that inhibiting mitochondria in BTICs may be a valuable strategy to decrease the progression of the disease [107]. Notably, the proliferation of stem-like cells in breast epithelium has been linked to the appropriate fragmentation and segregation of the mitochondrial population and the efficient preservation of the mitochondrial network. It should be noted that stem cells asymmetrically divide into one daughter cell that keeps stemness characteristics and another cell that is subjected to a differentiation program to contrast tissue aging and promote regeneration. Katajisto et al. showed that stem cells sort mitochondria using age by examining the destiny of old and young organelles during stem cells' asymmetrical division in the human breast epithelium. In addition, aged mitochondria are distributed asymmetrically among daughter cells by stem cells, with cells getting younger mitochondria being destined to keep stem features. To do this, stem-like cells use a highly effective method that includes mitochondrial spatial segregation. The loss of stem characteristics in the progeny cells may result from disrupting such carefully controlled processes during mitochondrial fission [108]. Following these findings, it has been shown that activating many oncogenic pathways, such as MAPK, contributes to mitochondrial fragmentation, which can be seen as an initial stage in reprogramming cells to become pluripotent [109]. Similarly, it has been demonstrated that c-Myc stimulates mitochondrial fusion in breast cancer cells to enhance clonogenic development, a characteristic of cells with stem characteristics. Notably, a mitochondrial retrograde signaling pathway has been demonstrated to initiate an EMT-like reprogramming, leading to altered morphology and increased migratory and invasive potential in human mammary epithelial cells. Maintaining a healthy mitochondrial population is required for maintaining and propagating the stem traits, so targeting these organelles in a therapeutic setting may represent a valuable strategy to eradicate CSCs. Based on these observations, mitochondrial functions and energetic dynamics may be involved in CSC propagation [110, 111]. On the other hand, mitochondria-dependent metabolism and OXPHOS could remain active and a source of energy supply in CSCs. Evidence indicated the usage of mitochondrial-dependent metabolism, increased mitochondrial ROS, and mass oxygen consumption in CSCs in Lung cancer [112], glioblastoma [97], Papillary Thyroid Carcinoma [113], Leukemia [114], ovarian cancer [115], and breast cancer [116]. It seems that the required ATP from OXPHOS plays an essential role in the metastatic and invasive properties of CSCs, which implies the possible role of mitochondria in CSCs [117, 118]. Additionally, ROS generation, which is mediated by mitochondrial metabolism, could take part in the progression of tumor and malignancy transformation [119]. Moreover, the increased mitochondrial mass has been located in invasive CSCs linked to chemotherapy resistance [120]. The activation of peroxisome proliferator-activated receptor-gamma co-activator one alpha (PGC1α), whose overexpression has been found in turmeric cells and the reduced stemness of breast CSCs is associated with the inhibition of this factor, seems to take a role in high mitochondrial metabolism in cancer cells [99, 121, 122]. The vulnerability of CSCs to treatment with inhibitors of OXPHOS has been illustrated in different studies, so far the repression of self-renewal and stemness properties and substantial temporary tumor growth/formation was observed during treatment with metformin as an inhibitor of the OXPHOS complex I [103, 123–125]. Therefore, mitochondrial metabolism can be a target to eliminate CSCs. There is raising evidence that not only CSCs take a balance between glycolysis and OXPHOS to make use of both of them, but also glutamine and lipid metabolism are intertwined in the metabolism of these cells. Glutamine takes part in the preparation of elements like amino-nitrogen and carbon, which are subsequently used in nucleotide, amino acid, and lipid production during the shortage of glucose to provide the energy needed for CSCs [126–128]. Furthermore, the role of lipids in the construction of cell membranes, energy consumption, and signaling transduction modifiers should be considered as a part of CSCs metabolism [129, 130]. Accumulation of unsaturated lipids like monounsaturated FAs (MUFAs) in CSCs has been confirmed by several studies. Stimulation of pathways involved in stemness was mediated by the enzyme stearoyl-CoA desaturase implicated in lipid desaturation. De novo through FA synthase also helps the survival and preservation of the properties of CSCs and is counted as a curial factor for recurrence and metastasis of the tumor [131–133]. In a recent study, it has been demonstrated that tyrosine kinase inhibitor (TKI) resistance in non-small cell lung cancer (NSCLC) could be caused by mutated epidermal growth factor receptor (EGFR) which uses the regulation of the fatty acid synthase (FASN) to induce TKI [134]. Taken together, it seems this capacity of switching between different metabolism statuses depending on the needs has made CSCs a challenging target to study, in light of the hypothesis of the plasticity of these cells.

#### Other metabolic sources for CSCs

The metabolic examination of CD133^+^/CD49f^+^ cells selected from hepatocellular carcinoma (HCC) revealed that liver-derived CSCs utilize fatty acid oxidation. In CD133^+^ cells isolated from CRC patients, there was an increase in lipid content and Wnt/B-catenin activity. In CSCs isolated from ovarian cancer patients, fatty acid oxidation-related genes were up-regulated. Etomoxir, a carnitine palmitoyltransferase-1 inhibitor, has also been demonstrated to limit spheroid formation in breast cancer in vitro and reduce tumor growth in vivo by blocking fatty acid oxidation [93, 135–137]. On the other hand, the expression of CSC markers and the efficiency of sphere formation have been demonstrated to decrease when fatty acid synthesis is inhibited by Soraphen A, cerulenin, and resveratrol [138–140]. However, more research is necessary to fully understand the function of lipid metabolism in CSC biology, especially in response to unique alterations in the tumor microenvironment. In contrast to glycolysis and OXPHOS, CSCs have been demonstrated to increase the PPP, especially during hypoxia and reoxygenation. Furthermore, rapid oxygenation increases the production of essential PPP enzymes, while hypoxia decreases it and causes the expression of glycolytic genes. This relationship between the activation of glycolysis and the PPP pathway in a microenvironment with various oxygen saturations may be due to cell migration being driven by glycolysis in hypoxia and cell proliferation being mediated by PPP in acute oxygenation. The function glutamine metabolism plays in CSCs from various malignancies, such as ovarian, pancreatic, and lung cancer, is notable [94, 141, 142]. In c-Myc-overexpressing cells, glutamine metabolism appears crucial, indicating that a pluripotency gene profile favors glutamine dependency. It has been demonstrated that inhibiting glutamine availability decreases the stemness gene signature and makes pancreatic CSCs more susceptible to radiation therapy in vitro and in vivo. In line with these findings, a related study using a mouse model of systemic metastasis revealed that blocking glucose metabolism with the glutamine analog L-DON can prevent the spread of metastatic disease to the liver, lung, and kidney [142–144]. The tumor microenvironment plays an important role in the progression of all types of cancer through the stages of sub-invasion and metastatic spread. Several studies have investigated the relationship between hyaluronic acid (HA) receptors and cancer cells. HA is a key extracellular component that helps control and regulates cell adhesion, migration, and invasive proliferation. CD44 is a major cell surface receptor for hyaluronic acid, a major component of extracellular matrices. Interactions of HA with its binding proteins CD44 are important in promoting tumor progression [145]. Many cancer cells are known to overexpress HA receptors such as CD44. After uptake by cancer cells, HA is broken down into low molecular weight components by hyaluronidase through CD44 receptor-mediated endocytosis. CD44 receptor overexpression was shown in various cancer cells, including colon, ovarian, breast and squamous cell carcinoma [146]. High levels of CD44 mRNA and protein expression levels in breast cancer are associated with significantly worse overall survival [147]. Many studies in recent years have identified the role of CD44 in a subpopulation of tumor cells with self-renewal capacity, the so-called CSCs [146]. A number of clinical studies have shown an association between CD44v6 expression and tumor progression in various tumor types. Günthert et al. [148] showed a significant correlation between CD44v6 expression and lymph node metastasis, lymphatic invasion when they transfected CD44 or CD44v6 expressing plasmids into non-metastatic rat pancreatic cancer cells. In addition, Wang et al. indicated a significant correlation between CD44 expression and stage, tumor size, and lymph node metastasis of gastric cancer. CD44v6 was related with *lymph node* metastasis, lymphatic invasion, and venous invasion [149]. Furthermore, various studies showed that increased expression of CD44 or CD44v6 was found in gastrointestinal tumors and was associated with tumor invasion, lymph node metastasis, and patient survival [149]. Wu et al. investigated the biological role and regulation of HA and its receptors in human gastrointestinal cancers. In their study a correlation between HA accumulation and tumor progression has been demonstrated in various gastrointestinal cancers. HA and HA fragment-tumor cell interactions can activate downstream signaling pathways, increase cell proliferation, adhesion, migration and invasion [150]. In another study, Li et al. carried out a study on the expression of hyaluronan receptors CD44 in stomach cancers. The results of their study shown that among CD44 isoforms, v6 is more related to malignant transformation of gastric epithelium. Expression of receptor for hyaluronan-mediated motility (RHAMM)*,* especially the cell surface variants, is closely correlated with tumor progression (*P*-value < 0.01) [145]. The CD44 expression may be mutually beneficial for gastric cancer cell invasion and metastasis. The roles of hyaluronic acid, hyaluronidases and HA receptors in cancer biology is complex and mediated by HA receptors expressed in cancer cells. Hence, HA was proposed as a drug carrier or for designing nanoparticles or liposomes for biocompatibility, biodegradability and based on the ability of CD44 to internalize HA [151]. Degradation of HA across a wide range of molecular sizes is stimulated by tissue ROS and Hyals that are found abundantly in tumor microenvironments. In particular, overexpression of Hyal-1 and Hyal-2 during cancer metastasis has been reported in many in vitro and in vivo studies, and it has recently been suggested that HA fragments promote cancer progression through *Hippo-Yap* signaling. However, the role of Hayl-3 in cancer progression is controversial as some studies have shown inhibition of tumor growth, while others have reported increased levels of the molecule in some solid tumors [151]. Clinicopathological analyzes show a strong correlation between increased expression of HA and Hyaluronan Synthase 2 and decreased expression of Hyal1 in tumor cells and poor survival in pancreatic ductal adenocarcinoma patients. Serum HA is known as a biomarker for liver fibrosis and cirrhosis, and its concentration is easy to determine clinically. However, little is known about the prognostic value of serum HA levels in patients with hepatocellular carcinoma [150]. Mima et al. reported that high preoperative serum HA levels (100 ng/ml or higher) in hepatocellular carcinoma patients independently predicted poor prognosis after hepatectomy [152]. The results obtained from these studies confirmed the results of the present study.

### The effects of mechanical forces on CSCs

**Fig. 2:**
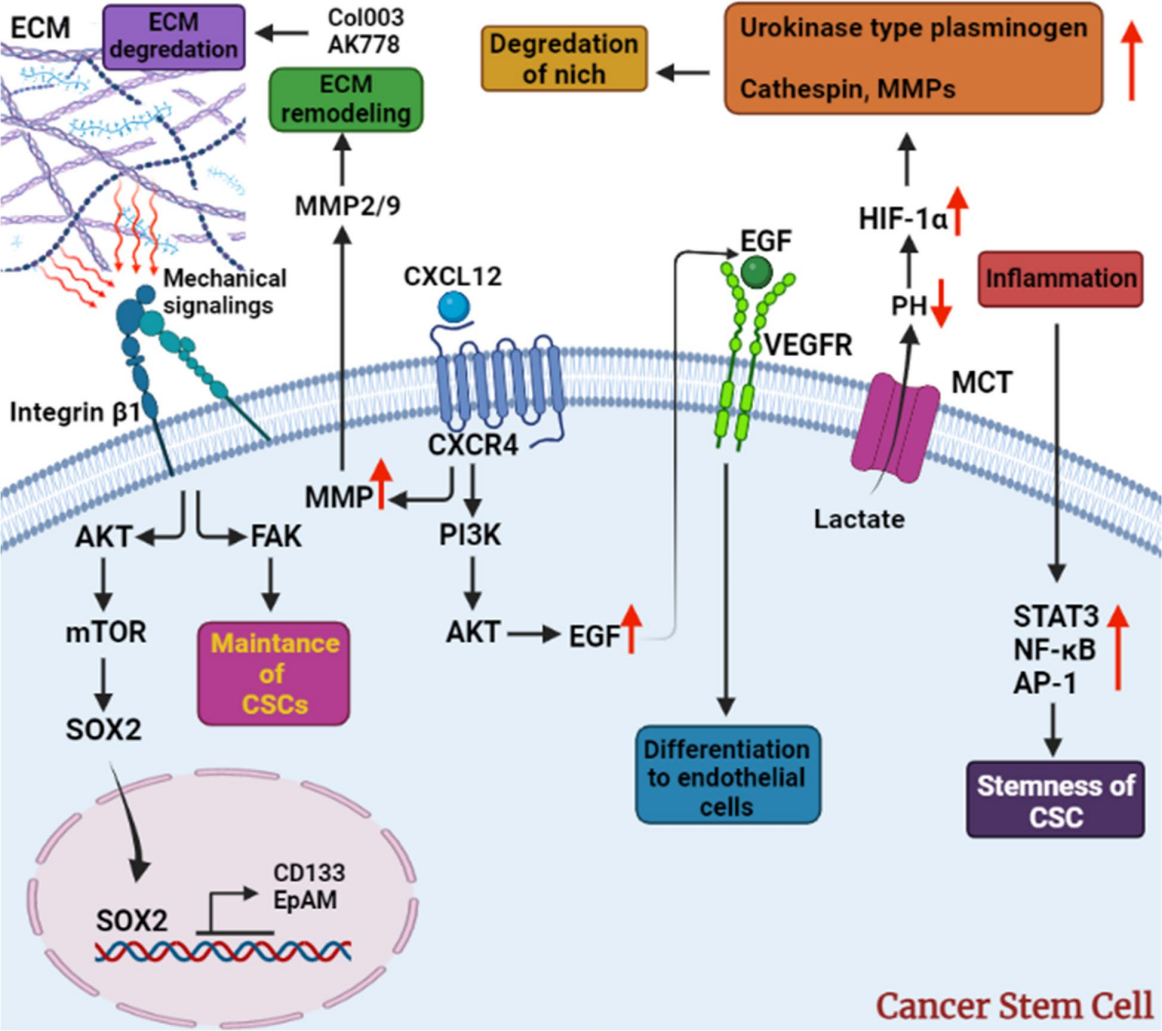
The illustration indicates interactions between CSCs and their environment: mechanical and chemical forces have their roles in this environment. For instance, inflammatory factors cause evaluation of STAT3, NF-κB, and AP-1 that result in stemness of CSCs. Sometimes, some changes in CSCs cause alterations in ECM that have indirect effects on CSCs like lactate emission from CSCs to ECM decreases environmental PH and then increases HIF-1α that results in elevation of urokinase-type plasminogen, cathepsins, and MMPs. Elevation of these substances causes degradation of the niche ( that is considered a mechanical change itself). On the other hand, CXCR4/CXCL12 axis triggers the PI3K/ AKT signaling pathway that evaluates EGF level in CSCs that binds to VEGFR and causes differentiation of CSCs to endothelial cells. Moreover, CXCR4/CXCL12 axis increases the MMPs level, which is an ECM remodeling factor in its own way. Mechanical forces (or signalings) from ECM are felt by integrin-β1 that starts FAK (maintenance of CSCs) and AKT/mTOR/SOX2 (expression of stemness markers such as CD133 and EpAM) signaling pathways

Carcinogenesis is related to interactions between tumor cells and mechanical stress in the TME. High mechanical stress in tumors can change a cancer cell's metabolism, behavior, and capacity to create cancer stem-like properties, accelerate the growth of the primary tumor, and encourage metastasis [153]. Mechanotransduction transforms mechanical signals into biochemical signals that activate the signaling pathways associated with tumorigenesis [153]. Moreover, Biomechanical signaling in TME has an important effect on stemness fate of cancer cells and CSC differentiation. Matrix stiffness and fluid shear stress are two examples of the most important mechanical forces that cause differentiation, migration, invasion, proliferation, EMT and so on by inducing different signaling pathways. According to studies, the extracellular matrix (ECM) in TME has more stiffness than normal tissues. This is due to the fact that cancer cells have a great growth power and more cells are accommodated in the limited environment, and also extracellular components such as collagen and proteoglycans are overexpressed in this space [154, 155]. Many studies have investigated the effects of stiffness of ECM on the stemness of cancer cells. For example You et al. [156] found that stiff ECM in HCC causes the transmission of mechanical signaling through integrin β1 molecule into the cell. The family of integrins are converters of environmental mechanical forces into chemical signaling [156]. Sensing the stiffness of the environment by integrin β1 activates the AKT/ mammalian target of rapamycin (mTOR)/SOX2 signaling pathway [156]. SOX2 is a factor that maintains cell stemness and causes the expression of stem cell characteristics factors such as CD133 and EpCAM [156]. FAK is another membrane signaling factor that is activated by integrin β1. FAK usually activates many signaling pathways including phosphoinositide 3-kinases (PI3K), mitogen-activated protein kinase (MAPK)/extracellular signal-regulated kinase (ERK) and cyclin D (Fig. 2). It has also been proven that the stemness of the CSCs and the initiation of cancer cells through the maintenance of CSCs can be strengthened by this factor [157, 158]. To that end, ECM stiffness has a very special role in tumorigenesis and stemness of cancer cells, therefore, agents have been used to disrupt tumor ECM in several studies [159]. Hsp47 is a chaperone in the ECM space that helps collagen folding, secretion, and assembly and can be a good target for disrupting tumor ECM [159]. Substances such as transforming growth factor beta (TGF-β) inhibitor (TGF-β induces expression of Hsp47), AK778, Col003, and methyl 6-chloro-2-oxo-2,3-dihydro-1,2lambda ~ 4 ~ , 3-benzothiazole-4-carboxylate are among these agents that inhibit Hsp47 activity [159]. TME is a hypoxic space and many tumorigenic factors are secreted by tumor cells in this environment, and as a result, a wide capillary network is formed in TME. However, this capillary network does not have appreciable efficiency and high permeability and there is a high interstitial fluid pressure in TME. In addition, another result of these events is a relatively extensive lymphatic system in the TME. The presence of IFP and the lymphatic system creates a weak mechanical force called fluid shear stress (FSS) [154]. Although the flow of this liquid and the strength of this force is weak, it has an important effect on the biological fate of CSCs. For example, FSS causes the differentiation of CSCs in lung cancer following the activation of the Wnt/β-catenin signaling pathway [160]. In addition, according to U. Triantafilluet al's research, FSS increases CSC in breast cancer [161] (Fig. 2).

#### Epithelial-mesenchymal transition

**Fig. 3:**
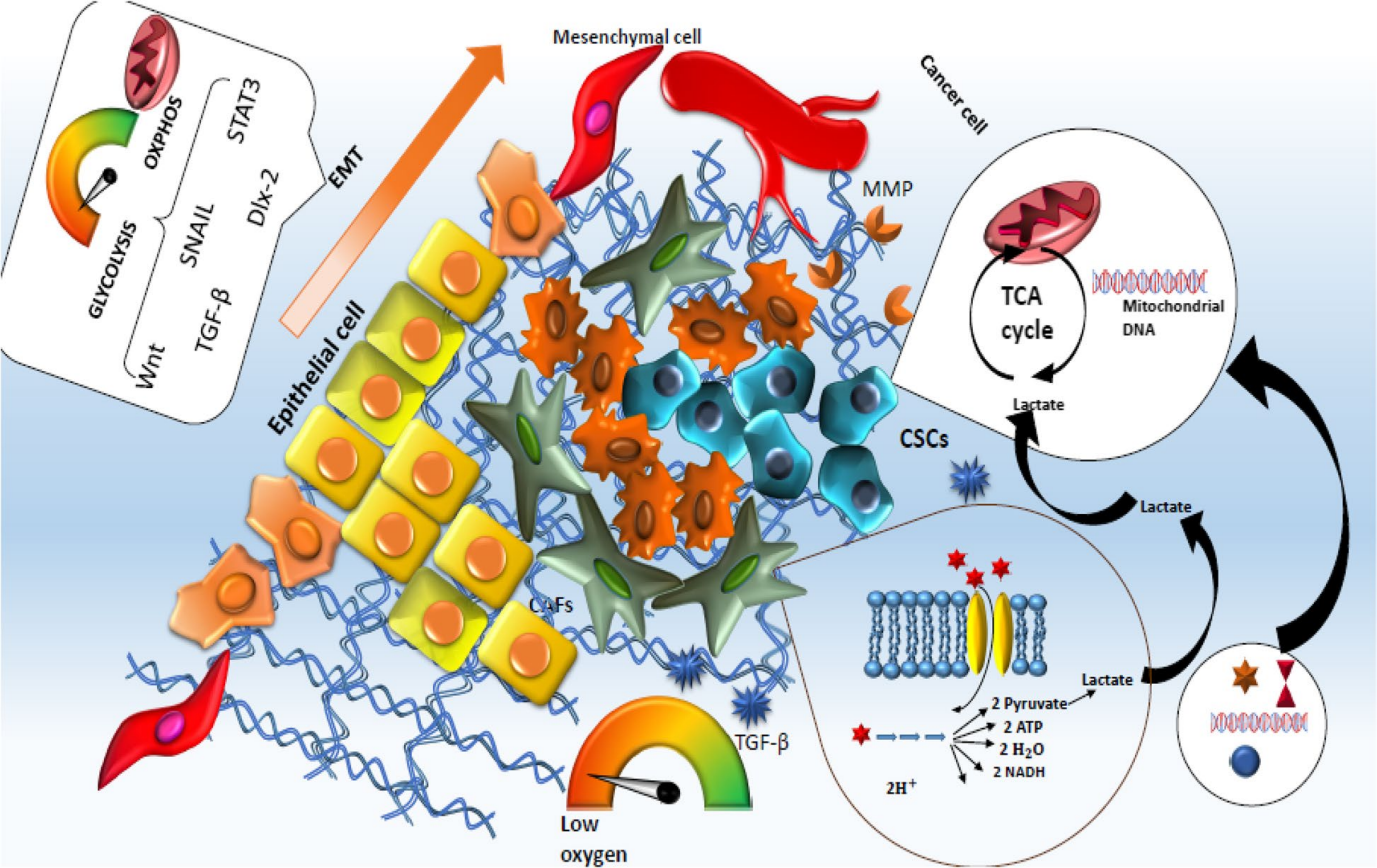
At the base of CSCs production, the EMT mechanism is involved and is associated with a decrease in mitochondrial activity and an increase in glycolysis. Signaling pathways implicated in EMT, such as Wnt/β-catenin, are also involved in the stimulation of stemness characteristics and the acquisition of CSCs. Regulation of metabolic adaptation can occur with the help of molecules involved in EMT such as Wnt and AKT, STAT3, Snail, HIF-1α, TGF-β, and Dlx-2. Cancer-associated fibroblasts (CAFs) induce tumor remodeling through the release of factors such as matrix metalloproteinases (MMPs) and enzymes, and angiogenesis that summons other inhibitory cells, growth, and metastasis. Lactate released by CAFs following glycolytic metabolism in these cells is taken up and used by CSCs such as epithelial cells to supply the TCA cycle, which in turn promotes processes such as metastasis, self-renewal, and invasion of MSCs provide

Initial steps of metastasis of turmeric cells begin with EMT and involve epithelial cells losing their identity, changing morphologically and acquiring mesenchymal cell properties [162, 163]. This hijacked process by cancer cells is initially used for homeostasis, development of organs and tissue healing and consists of a series of transcriptional changes. Some of the involved factors in this process are HIF-1α, Twist-related protein 1/2, distal-less homeobox 2 (Dlx-2), Snail, zinc finger E-box-binding homeobox (ZEB) 1/2, and Slug [163–166]. The EMT, as the center of tumor malignancy, is closely correlated to CSCs [29, 167, 168], because it seems that at the base of CSCs generation, the EMT mechanism is involved and is linked with decreased mitochondrial activity and enhanced glycolysis [164, 169]. Moreover, signaling pathways imprinted in EMT like Wnt/β-catenin are also involved in the stimulation of stem-ness features and acquisition of CSCs [170]. More interestingly, regulation of metabolic adaptation can occur with the help of molecules involved in EMT like Wnt and AKT, STAT3, Snail, HIF-1α, TGF-β, and Dlx-2 (Fig. 3) [63, 171–176]. Snail has been reported to be involved in the repression of mitochondria and supporting glycolytic metabolism [171, 174]. In EMT-derived cancer cells, STAT3 could promote glycolysis through positive regulation of transporters linked to anaerobic glycolysis [177]. Dlx-2, whose expression relies on the metabolic stress induced by ROS through the expression of SNAIL, contributes to the glycolytic switch and inhibition of mitochondrial activity mediated by TGF-β/Wnt signaling pathway [171]. By promoting EMT in breast cancer, overexpression of SNAIL could cause the resistance of breast cancer cells to the lysis induced by CD8^+^ T cells [178]. Additionally, negative regulation of mitochondrial function induced by HIF-1α by promoting the activity of PDK has made this factor one of the central regulators of the glycolytic switch [173, 179].

#### Cellular plasticity

As one of the complications ahead of tumor therapy, turmeric cells' plasticity is defined as their ability to swing between asymmetric division and symmetric division, CSCs and non-CSCs, quiescence, and proliferation [180, 181]. The dynamic conversion property of CSCs has been attributed to the emergence of drug resistance [182, 183]. Plasticity can make the heterogeneity of tumor worse and eradication of tumors more complicated as CSCs can convert from quiescence to proliferative or vice versa, making it possible for the recurrence of cancer years after chemotherapy [184]. The network between CSCs, surrounding cells, the niche of CSCs, and four intrinsic factors play a vital role in the regulation of plasticity by providing the agents and necessary signals in a wide range of forms [185, 186]. 1) The acidic microenvironment of a tumor contributes to the reprogramming of non-CSCs and their dedifferentiation of them [187]. 2) The inflammatory microenvironment could promote the recruitment of inflammatory cells and the release of factors that favor CSCs resulting in the activation of signaling pathways like Wnt, imprinted in de-differentiation [188–190]. 3) Metabolic adjusting, which was discussed in the previous section, is one of the central regulators of plasticity as a regulator of conversion from glycolysis to OXPHOS or vice versa [103]. 4) Hypoxic conditions of TME can also promote the induction of treatment resistance in CSCs and stemness [191].

#### CSCs and tumor angiogenesis

Angiogenesis has been recognized to be vital for tumor growth, metastasis, and migration. Vascularization can happen in different ways in the tumor, such as sprouting angiogenesis, recruitment of endothelial progenitor cells, intussuscepted angiogenesis, vascular mimicry, and trans-differentiation of CSCs [192]. CSCs, besides their capability to differentiate to ECs, by secretion of factors such as VEGF, HIF-1α, and CXCL12, can promote the recruitment and migration of ECs and mesenchymal stem cells (MSC) to the niche of tumor and differentiation of them into ECs [193–195]. In this regard, high expression of MMP-2 and MMP-9 by CSCs contributes to the initiation of Extracellular matrix (ECM) remodeling. Moreover, the over-expression of VEGF receptors in these cells compared to their partners in angiogenesis is notable (Fig. 3) [196, 197]. On the other hand, the autocrine secretion of VEGF can be stimulated by high expression of CXCR4 and its ligand and activation of the downstream pathway through the PI3K/AKT signaling pathway, which eventually influences the expansion of CSCs via stimulation of stimulating neuropilin-1 [197, 198]. Finally, the transformation of ECs to endothelial progenitor cells seems to be promoted by the expression of Notch and vascular–endothelial cadherin (VE-Cadherin) involved in this process [196, 199–201].

#### CSCs and tumor invasion

Invasion is considered the first step of metastasis and migration to nearby or faraway organs. This process involves a cascade of steps that enables cells to pass through the tissue structures and vessel walls. Different models of migration are used in different malignancies such as amoeboid migration, mesenchymal migration, and collective cell migration [202]. The invasion begins with the loss of cell–cell adhesion mediated by the loss of specific proteins involved in adhesion, which also influences cell–matrix adhesion. Moreover, the ability to change and deregulate the surrounding extracellular matrix via matrix metalloproteinase is the next vital step to migration. In the next levels, named intravasation and extravasation, cancer cells pave the way to enter the blood vessels or lymphatic system, and after exiting the circulatory system, inhabit organ parenchyma and start colonization [203]. The release of TGF-β to support the cellular invasion and metastasis by CSCs is another way through which CSCs promote invasion [204–206]. Furthermore, the premetastatic niche (PMN) is the other part where CSCs could function as metastasis players. TGF-β and VEGF, which can be released by CSCs, enhance vasculature permeability, CCL-9, and inflammatory agent secretion and enhance the utilization of bone marrow-derived cells (BMDCs) [207–210]. CXCL-12, which plays an essential role in metastasis, angiogenesis, and MMP expression and protects its activity in TME, is also expressed in CSCs [211, 212]. Aerobic metabolism of glucose by cancer cells in TME results in the formation of lactate, which is lethal to normal cells, while cancer cells, through the expression of MCTs transporters, transfer this substance to their niche, leading to decreased pH of TME [79]. The acidity of the environment provided by lactate, which itself can stimulate HIF-1α, is the favorite factor of MMP, cathepsins B, L, D, and urokinase-type plasminogen activators to function better and facilitate the degradation of the surrounding niche [213–216]. More interestingly, phosphoglucose isomerase (PGI), as one of the enzymes involved in glycolysis, has been noted to act as an autocrine motility factor (AMF) with an anti-apoptotic effect. PGI is used for enhancing metastasis, invasion, and cellular migration through different mechanisms like upregulation IL-8 secretion, which is imprinted in migration, induction of EMT by promoting the expression of mesenchymal markers expression in epithelial cells and halting expression of epithelial markers [217–220]. PGI has been observed to be capable of inducing self-renewal properties and promoting tumorigenesis in glioma CSCs [221]. PKM2 as one of the other vital enzymes in glycolysis, has been correlated with the decrease of E-cadherin and promoting the signaling pathway of EGFR and, as a result, cellular migration [222]. Additionally, this enzyme has been observed to be able to promote the induction of cancer stem-like cells [223]. Lactate dehydrogenase A (LDHA), as the key element of converting pyruvate to lactate, overexpressed in tumor cells could also contribute to the regulation of TGF-β and the rise of MMP-2 in glioma cells [224]. In addition, in another study, breast cancer stem-like properties were generated via an LDHA-dependent way [225]. Taken together, it is reckoned that CSCs and cancer cells' metabolism at the time of hypoxia, which mostly turns to aerobic glycolysis, and acidification facilitate the initiation of invasion and migration to other niches.

### CSCs and tumor microenvironment

The behavior of turmeric cells, including CSCs, is highly defined by the presence of heterogenic cell types, blood vessels, lymph vessels, ECM, and signals received from the microenvironment surrounding them. The tumor niche can be responsible for the creation of a web that defines the response of the immune system, induction of CAFs, MSCs, ECs, ECM, and soluble factors [226]. ECM with a unique composition contributes to impacting the signaling, cellular movements, invasion, and angiogenesis [227, 228]. Not only ECM acts as a blocking wall-facing agent used in chemotherapy and radiotherapy, but it also participates in the creation of hypoxia [229, 230]. Growth factors and other soluble agents existing in TGF-ß, interleukin-6, fibroblast growth factor (FGF), and hepatocyte growth factor (HGF). In addition, the protection of tumor growth and induction of resistance to therapies [231]. Some of the critical cell types present in the tumor microenvironment are Immune cells like TAM, natural killer (NK) and dendritic cells (DCs), T lymphocytes and B lymphocytes, and Myeloid-derived suppressor cells (MDSC), fibroblast cells, which could form a network to benefit the tumor cells or fight against them [232]. More importantly, the dominant condition of TME could also define invasion and progression. Inflammation and hypoxia are two vital situations needed for the growth of the tumor [233]. The mutation rate needed for the initiation of a tumor and the proliferation rate can be accelerated in an inflammatory environment [234, 235]. The production of ROS, produced by the inflammatory immune cells recruited to the location of inflammation, contributes to DNA damage, which could also lead to the arrest of the mismatch repair system [236]. The cytokines, chemokines, and growth factors released in the process of inflammation contribute to the activation of transcription factors like STAT3, nuclear factor-kappa B (NF-κB), and AP-1 imprinted in cellular proliferation and the induction of reprogramming needed to gain stemness and self-renewal properties [237] suggesting that there must be a link between the CSCs and chronic inflammation. IL-6, as one of the factors mostly found during the incidence of inflammation, is vital for the survival of CSCs, as the exposed cells to this factor gained an increased capacity for invasion and resistance [238]. More interestingly, it seems that CSCs tend to firm their own niche in different ways [239]. This cross-talk between CSCs and TME through the metabolites, exosomes, cytokines, growth factors, and chemokines, including vascular endothelial growth factor (VEGF), HIF-1, matrix metalloproteinases (MMPs), CCL5, CCL2, TGF-β, IL-1β, IL-8, and IL-6 [240–245] can be the defining factor in the regulation of the processes such as metastasis, angiogenesis, immune escape and drug resistance [246].

#### TME contributes to CSCs metabolism

The surrounding niche of CSCs is one of the defining factors that could explain the plasticity of these cells' metabolism. Factors like CAF, endothelial cells, inflammatory agents, the presence of immunomodulatory cells, and conditions like inflammation and hypoxia could be considered as determining factors in the metabolic switch in CSCs [57, 247]. For example, activation of NF-κB causes to release inflammatory cytokines like IL-1β, IL-6, and IL-8 can promote the recruit of AKT and PI3K and subsequently contributes to the self-renewal property of CSCs [248–251]. A high rate of ketone bodies and lactate, which represent the usage of glycolysis and ketogenesis in TME, in the companionship of factors like TGF-β and HIF-1α, could contribute to the induction or preservation of stemness in CSCs [252–255]. In the following parts, the role of each of the mentioned factors will be discussed. Simultaneous high-speed proliferation and lack of enough blood vessels put most solid tumors in a hypoxia condition [256], resulting in the induction of HIF. The hypoxic condition of the tumor can be responsible for the alteration in TME like EMT, suppression of apoptosis, metabolic changes, invasion of the tumor, infiltration of modulatory cells, and production of modulatory agents, and neovascularization [257–259]. In addition, to the fact that hypoxia can support stemness and undifferentiated properties. The potential to enhance glucose transporters on the surface, and shift to glycolysis, facing the lack of nutrients and hypoxia, represents the high adaptability of these groups of cells [260, 261]. Moreover, in a study, an ensconced number of breast CSCs as a consequence of the production of HIF-1α and activation of AKT/β-catenin was observed after the generation of intratumoral hypoxia with the help of anti-angiogenic agents [262, 263]. Not surprising that in xenograft models of breast cancer, inhibition of HIF-1α was accompanied by a decreased population of breast CSCs [264–266]. In NSCLC, hypoxia also resulted in gefitinib-Resistant Lung CSCs enrichment, and the expansion of this population was mediated by insulin-like growth Factor 1 Receptor (IGF1) [267]. In glioma stem cells, enhanced activity of this population was observed under hypoxic conditions; additionally, suppression of HIF-1α and HIF-2α by shRNA caused a decline in the activity of CSCs [268]. Moreover, enhancement of stem-ness property in glioma and leukemia CSCs because of HIF-1α activation has been reported [269, 270]. Finally, the formation of CSCs seems to be under the influence of HIF-2α, which can stimulate the activation of c-Myc by controlling the expression of OCT-4 [271].

#### CSCs and CAFs

as the first assistance of CSCs which can be trained and reprogrammed to obtain a protumorigenic character, in the companionship of other suppressor cells by secreting growth FGF, HGF, and CXCL12, accelerating the tumor As the predominant population in solid tumors, fibroblast cells, named CAF are recruited during the healing process after a sustained inflammation. These cells are considered growth and metastasis via induction of tumor remodeling via the release of factors like MMPs, enzymes, and angiogenesis summoning other inhibitory cells [226, 272, 273]. Moreover, the protection fence provided by these cells could facilitate the therapy resistance and recruitment of immune cells to secret inflammatory factors, which makes the environment ready for tumor progression besides suppressing activated lymphocytes [272, 274, 275]. Moreover, supporting the invasive phenotype of CSCs by CD90^+^ CAFs has been observed following the habitation of CD44^+^ CD90^+^ CSCs at periphery sites of breast tumors, where they were in direct contact with CAFs [276]. In addition, their role in the regulation of CSCs metabolism has been noticed. Reprogrammed and dependent CAFs on aerobic glycolysis can also gain energy from autophagy to fuel the processes such as migration, proliferation, and cytokine secretion [277–279]. In this line, the usage of autophagy in CAFs could provide the demands of pancreatic ductal adenocarcinoma [280]. In breast cancer cells, enhanced expression of cell membrane-bound GLUT-1 transporter increasing the uptake of glucose is promoted by the secretion of cytokines by CAFs [281]. The usage of glutamine in CAFs, which consequently supports the tumorigenicity and microenvironment of CSCs, is a perfect target to stop the development of ovarian tumors [282]. Moreover, metabolic reprogrammed CAFs in NSCLC was correlated with a rise in the glycolytic metabolism of tumor [283]. Alternatively, in some experiments, it has been revealed that the metabolites produced in CAFs during a phenomenon called "reverse Warburg Effect" can be consumed by the CAFs surrounding cells, leading to enhanced tumorigenicity [284]. Moreover, the transference of mitochondrial DNA via exosomes from CAFs to breast CSCs promotes OXPHOS and possible therapy resistance in an OXPHOS-dependent manner [285], which provides evidence that CSCs show plasticity in their metabolism. Lactate released by CAFs, following the glycolysis metabolism in these cells seems to be absorbed and used by epithelial CSC-like cells to fuel the TCA cycle, which in return fuels processes such as metastasis, self-renewal, and invasion in CSCs [286].

#### CSCs and ECs

ECs are one of the vital parts of TME that orchestrate with other cells to provide tumor progression, which can be recruited to the niche of the tumor upon the section of VEGF, HIF-1, and SDF-1/CXCL12 by CSCs to initiate the process of angiogenesis [24, 287, 288]. Co-culturing CSCs and ECs have revealed that ECs play an essential role in supplying the factors required for the maintenance of self-renewal and stem-ness in CSCs [289]. In glioma and colorectal cancer, robust self-renewal and stemness of CSCs through the Notch signaling pathway was attributed to the presence of ECs [290, 291]. In breast cancer, the activation of NF-κB in CSCs, which led to the secretion of S100A8/9 and establishment of resistance to doxorubicin and cyclophosphamide, was a result of the production of TNF-α by ECs [292]. Activation of STAT3 by the IL-6 secreted in head and neck squamous cancer cells in tumor-associated endothelial cells has nominated this factor to be a part of CSCs and ECs network, considering the role of IL-6 in the induction of glycolytic pathway, as a glycolytic phenotype in ECs cells [293, 294]. Studies have introduced glycolysis as the primary source of energy in ECs, and PFKFB3 knockdown in ECs, resulting in sabotaging the glycolytic pathway, has caused a reduction in angiogenesis [293]. Cancer-associated endothelial cells (CAEC), under the influence of the accumulated lactate in TME, which results in enhanced IL-8/CXCL-8 signals, can support angiogenesis [294]. Although aerobic glycolysis has been recognized as the favorite source of energy in ECs [295], stimulation of signaling pathways imprinted in preserving the prevascular niche has been correlated with mitochondrial activity in ECs. As one of the crucial factors in angiogenesis, VEGF has indicated the capacity to promote mitochondrial metabolism in ECs, which could respond to the expansion of CSCs [278, 296, 297].

### CSCs and Immunomodulation

Low concentration of nutrients plus high speed expanding cancer cells starving for glucose and the mass of metabolites left from turmeric cells metabolism make TME like a barrier that stops immune cells from getting fully functional. In particular, low levels of oxygen or hypoxia could lead to enhanced pyruvate dehydrogenase kinase and lactate dehydrogenase A expression, which subsequently sabotage mitochondrial respiration and ROS production and shut down the conversion of pyruvate to lactate, respectively. Deprivation of glucose seems to cause a reduction in aerobic glycolysis and the regulation of NFAT signaling, which influences and halts the functions of T cells [298]. Lactate besides protons (H^+^), which are transported to the niche of turmeric cells, are among the most abundant metabolites released by these cells [299–301]. The accumulated lactate leads to low pH in the TME or acidification, which limits the activity of immune cells by suppressing the activation of NK cells and confining the production of IFN-γ, and induction of apoptosis in T and NK cells [298, 302, 303]. On innate immunity cells, like DC cells, remaining in the tolerogenic state, decreased expression of CD1, the release of IL-12 and enhanced secretion of IL-10, and diminished migration ability [304, 305]. Moreover, reprogramming of DCs mediated by TME could result in the formation of regDCs, capable of sabotaging antitumor activity and facilitating lung tumor invasion [306]. Concerning macrophage responses, there is some debate on the activity and properties of these cells in a lactic environment and polarization towards the M2 phenotype by stabilizing HIF-1α [307]. By stimulation of STAT3, M2 macrophages, which in a tumor are named tumor-associated macrophages, support population, invasion, and drug resistance of CSCs. Stimulation of self-renewal in CSCs can be promoted by the release of TGF-β, IL-10, and IL-6 by TAM [308]. Moreover, the cytokines released by macrophages have been linked to the EMT reprogramming by the downregulation of miR-138. The stemness in NSCLC could also be regulated by TAMs through the upregulated expression levels of ubiquitin-specific peptidase 17-like family member 9, which could result in the production of inflammatory factors, which consequently lead to increased stemness [309]. NK cells, which are known for their function in cytokine production and cytotoxicity, play a vital role in immunity against cancer [310]. From taking part in the activation of CD8^+^ T cells and monocytes, helping maturation of DCs, defining polarization of T cells to production of tumor necrosis factor-alpha (TNF-α), IFN-γ, and granulocyte–macrophage colony-stimulating factor (GM-CSF) and eradication of infected and tumor cells by two mechanisms of antibody-dependent cellular cytotoxicity, in the absence of prior stimulation through natural cytotoxicity [310, 311]. More notably, in targeting CSCs, NK cells have an essential role as a study has shown the increased susceptibility of CSCs to NK killing [312], which must be because of the decreased expression of MHC-I to protect their growth in TME [313]. T cells, categorized as tumor-infiltrating lymphocytes (TIL) in tumors in charge of regulating immune responses, consist of subtypes like Th2, and T-reg that could favor tumors, and in some cases could be recruited by turmeric cells, and groups like Th1 and CD8^+^ T cells act against cancer and mediates eradication of cancer cells [314, 315]. Studies on the effect of low pH of TME have reported that pH like 6.6 can put the function and expansion of T cells in jeopardy, plus decreased expression of TCR, IFN-γ, IL-2, TNF-α consistent with the mentioned changes [316, 317]. Taken together, it seems that the acidic microenvironment of the tumor, which is the result of the glycolytic metabolism of CSCs and cancer cells, could protect them from the attack of immune cells in different ways and help the invasion of CSCs.

### Therapeutic perspectives

Given the crucial role of CSCs in the development, invasion, and relapse of tumors, and their plasticity in the face of different conditions, choosing the most effective approach to eradicate these cells is challenging. Concerning the close relation between the self-renewal, stemness, and metastasis properties of CSCs and their metabolism, one of the proposed solutions is targeting the metabolism of CSCs. In xenograft models, experiments with a focus on mitochondrial respiration have led to the depletion of these cells via sensitization to chemotherapy. For instance, in metastatic melanoma, enrichment of JARID1B slow-cycling subpopulation was formed following the treatment with cisplatin and vemurafenib, inhibition of OXPHOS was then resulted in the suspension of JARID1B^+^ subpopulation formation and making melanoma cells more vulnerable to chemotherapy agents [318] (Table 1). In PDAC cells with mutations in KRAS, targeting OXPHOS with inhibitors like oligomycin, combined with therapies aiming at KRAS, showed better elimination rather than just using treatments specified for the oncogene [98]. The metabolic shift in CSCs of colorectal cancer is correlated with 5-fluorouracil (5-FU) resistance, and a combination of 5-FU and metformin to block mitochondrial activity has effectively reduced the population of CSCs [319]. In glioblastoma CSCs, combination therapy of 3-bromopyruvate that targets glycolysis and doxorubicin led to effective inhibition of tumor and elimination of CSCs [320]. In a similar experiment on pro-neural and mesenchymal CSCs, high radiation therapy resistance and invasion of mesenchymal CSCs were diminished with the use of an inhibitor of ALDH [321]. Moreover, since CSCs recruit a wide range of cells and factors in their niche to support their survival and growth, perhaps targeting these allies could facilitate the eradication of CSCs. For example, targeting VEGF via monoclonal antibody combined with CXCR4 antagonist in glioblastoma resulted in enhanced survival, and the use of POL5551 alone as the CXCR4 antagonist affected the existing CSCs [322]. Although the usage of anti-VEGF led to an increased breast CSCs, through induction of hypoxia [262], in the CSCs population of NSCLC, combination therapy of anti-hepatoma-derived growth factor (HDGF) antibody and VEGF tyrosine kinase inhibitor was successful in diminishing this population [323]. Blocking Hedgehog signaling in breast cancer led to the halter of the activity of CAFs, which subsequently made CSCs more susceptible to chemotherapy [324]. Treatment of CD10^+^ GPR77^+^ CAFs with anti-GPR77 antibodies was shown to diminish tumor formation and sensitize lung and breast cancer cells to chemotherapy [325]. Though developing new therapies based on CAFs is still going on, such as the therapies via CAR-T cell, SynCon DNA vaccine, and Oncolytic adenovirus focused on fibroblast activation protein (FAP) [326–329], targeting JAK/STAT3 pathway [330] or Blocking pan-TGF-β and GARP [331]. One of the CAFs is a type II transmembrane glycoprotein termed FAP. It was shown that when FAP-specific CAR-T cells were used alongside a tumor antigen-specific CAR, an enhanced anti-tumor activity in A549 lung cancer cells was observed [332]. A pioneering study has also shown oral administration of DNA-based FAP vaccine-induced CD8^+^ T cell-dependent killing of CAFs, which substantially increase the intratumoral uptake of chemotherapeutic drugs in multi-drug-resistant murine colon and breast carcinoma. Of note, FAP-specific CAR-T cell treatment in an immunocompetent mouse model has been shown to boost host immunity. Similarly, the co-introduction of anti-FAP and anti-tumor CAR-T cells has also shown to enhance anti-tumor immunity in xenografted immune-deficient mouse models [333].

**Table 1:** Therapeutic perspectives of targeting CSCs in the tumor microenvironment

Drug	Cancer/Cell line	Description	Refs
Cisplatin and vemurafenib	Melanoma	Enrichment of JARID1B slow-cycling subpopulation was formed Suspension of JARID1B^+^ subpopulation formation and making melanoma cells more vulnerable to chemotherapy agents	[302]
Oligomycin,	PDAC cells with mutations in KRAS	Better elimination of CSCs rather than just using treatments specified for the oncogene	[303]
5-fluorouracil and metformin	Colorectal cancer	Effectively reduced the population of CSCs by blocking the mitochondrial activity	[304]
3-bromopyruvate and doxorubicin	Glioblastoma	Effective inhibition of tumor and elimination of CSCs	[305]
VEGF monoclonal antibody and POL5551	Glioblastoma	Enhanced survival via affecting the existing of CSCs	[307]
anti-GPR77 antibody	Breast and lung cancer	Diminish tumor formation and sensitizing lung and breast cancer cells to chemotherapy	[310]

## Conclusion

CSCs as the initiator of tumors, involved in processes of metastasis, invasion, and therapy resistance have been paid attention to be capable of being potential targets for tumor therapy. Mounting experiments are now focused on the metabolic side of these cells, which gets important in the occurrence of phenomena like EMT, hypoxia, metastasis, and tumor growth, as the contradictory data on glycolysis or OXPHOX reliance, represents the involvement of other factors. Therefore, a better understanding of the plasticity and the metabolic state of CSCs in different stages of malignancies, and how the counterparts or enemies of CSCs get to affect this machinery is required so that we could get one step closer to developing new therapies to eliminate CSCs via targeting the metabolism of CSCs or the partners of CSCs in the TME.

## Data Availability

The datasets used and/or analyzed during the current study are available from the corresponding author on reasonable request.

## Abbreviations

CSCs: Cancer stem cells
EMT: Epithelial-mesenchymal transition
TME: Tumor microenvironment
TAM: Tumor-associated macrophages
CAF: Cancer-associated fibroblasts
EC: Endothelial cells
TICs: Tumor-initiating cells
AML: Myeloid leukemia
SCID: Severe combined immune-deficient
SOX2: Sex-determining region Y HMG-box 2
ALDH1A1: Aldehyde dehydrogenases 1A1
ABCG2: ATP-binding cassette sub-family G member 2
JAK/STAT: Janus kinase /signal transducer and activator of transcription
TCA: Tricarboxylic acid cycle
OXPHOS: Oxidative phosphorylation
PDK: Pyruvate dehydrogenase kinase
CRC: Colorectal cancer
RNAi: RNA interference
PFKFB4: 6-Phosphofructo-2-kinase/fructose-2,6-biphosphatase 4
PKM2: Pyruvate kinase M2 hypotype
HIF-1: Hypoxia-inducible factor-1
NADH: Nicotinamide adenine dinucleotide
iPS: Pluripotent stem cells
G6P: Glucose-6-phosphate
GLUT-1: Glucose transporter 1
HK-1: Hexokinase 1
ROS: Reactive oxygen species
OCR: Oxygen consumption rate
DRP1: Mitochondrial dynamics
PGC1α: Peroxisome proliferator-activated receptor-gamma co-activator one alpha
MUFAs: Unsaturated lipids like monounsaturated FAs
TKI: Tyrosine kinase inhibitor
FASN: Fatty acid synthase
HCC: Hepatocellular carcinoma
RHAMM: Hyaluronic acid (HA), hyaluronan-mediated motility
ECM: Extracellular matrix
mTOR: Mammalian target of rapamycin
PI3K: Phosphoinositide 3-kinases
MAPK: Mitogen-activated protein kinase
ERK: Extracellular signal-regulated kinase
TGF-β: Transforming growth factor beta
FSS: Fluid shear stress
Dlx-2: Distal-less homeobox 2
ZEB: Zinc finger E-box-binding homeobox
MSC: Mesenchymal stem cells
BMDCs: Bone marrow-derived cells
PGI: Phosphoglucose isomerase
AMF: Autocrine motility factor
PKM2: Pyruvate kinase isomerase M2
LDHA: Lactate dehydrogenase A
FGF: Fibroblast growth factor
HGF: Hepatocyte growth factor
NK: Natural killer
DCs: Dendritic cells
MDSC: Myeloid-derived suppressor cells
VEGF: Vascular endothelial growth factor
HIF-1: Hypoxia-inducible factor-1
MMPs: Matrix metalloproteinases
NF-κB: Nuclear factor-kappa B
IGF1: Insulin-like growth Factor 1 Receptor
CAEC: Cancer-associated endothelial cells
TNF-α: Tumor necrosis factor-alpha
TIL: Tumor-infiltrating lymphocytes
5-FU: 5-fluorouracil
HDGF: Anti-hepatoma-derived growth factor
FAP: Fibroblast activation protein

## References

[1] Bray F, Ferlay J, Soerjomataram I, Siegel RL, Torre LA, Jemal A (2018). Global cancer statistics 2018: GLOBOCAN estimates of incidence and mortality worldwide for 36 cancers in 185 countries. CA..

[2] Heng WS, Gosens R, Kruyt FAE (2019). Lung cancer stem cells: origin, features, maintenance mechanisms and therapeutic targeting. Biochem Pharmacol.

[3] Munro MJ, Wickremesekera SK, Peng L, Tan ST, Itinteang T (2018). Cancer stem cells in colorectal cancer: a review. J Clin Pathol.

[4] Parada LF, Dirks PB, Wechsler-Reya RJ (2017). Brain Tumor Stem Cells Remain in Play. J Clin Oncol.

[5] Yamashita T, Wang XW (2013). Cancer stem cells in the development of liver cancer. J Clin Investig.

[6] Wainwright EN, Scaffidi P (2017). Epigenetics and cancer stem cells: unleashing, hijacking, and restricting cellular plasticity. Trends in cancer.

[7] Deshmukh A, Deshpande K, Arfuso F, Newsholme P, Dharmarajan A (2016). Cancer stem cell metabolism: a potential target for cancer therapy. Mol Cancer.

[8] De Francesco EM, Sotgia F, Lisanti MP (2018). Cancer stem cells (CSCs): metabolic strategies for their identification and eradication. Biochem J.

[9] Mostafavi S, Zalpoor H, Hassan ZM (2022). The promising therapeutic effects of metformin on metabolic reprogramming of cancer-associated fibroblasts in solid tumors. Cell Mol Biol Lett.

[10] Bjerkvig R, Tysnes BB, Aboody KS, Najbauer J, Terzis A (2005). The origin of the cancer stem cell: current controversies and new insights. Nat Rev Cancer.

[11] Bu Y, Cao D (2012). The origin of cancer stem cells. Front Biosci Scholar.

[12] Friedmann-Morvinski D, Verma IM (2014). Dedifferentiation and reprogramming: origins of cancer stem cells. EMBO Rep.

[13] Basu AK (2018). DNA damage, mutagenesis and cancer. Int J Mol Sci.

[14] Blackadar CB (2016). Historical review of the causes of cancer. World J Clin Oncol.

[15] Hanahan D, Weinberg RA (2011). Hallmarks of cancer: the next generation. Cell..

[16] Reya T, Morrison SJ, Clarke MF, Weissman IL (2001). Stem cells, cancer, and cancer stem cells. Nature..

[17] Li L, Neaves WB (2006). Normal stem cells and cancer stem cells: the niche matters. Can Res.

[18] Perekatt AO, Shah PP, Cheung S, Jariwala N, Wu A, Gandhi V (2018). SMAD4 suppresses WNT-driven dedifferentiation and oncogenesis in the differentiated gut epithelium. Can Res.

[19] Oikawa T (2016). Cancer stem cells and their cellular origins in primary liver and biliary tract cancers. Hepatology.

[20] Afify SM, Seno M (2019). Conversion of stem cells to cancer stem cells: undercurrent of cancer initiation. Cancers.

[21] Bu P, Chen K-Y, Lipkin SM, Shen X (2013). Asymmetric division: a marker for cancer stem cells?. Oncotarget.

[22] Lapidot T, Sirard C, Vormoor J, Murdoch B, Hoang T, Caceres-Cortes J (1994). A cell initiating human acute myeloid leukaemia after transplantation into SCID mice. Nature.

[23] Schatton T, Murphy GF, Frank NY, Yamaura K, Waaga-Gasser AM, Gasser M (2008). Identification of cells initiating human melanomas. Nature.

[24] Ponti D, Costa A, Zaffaroni N, Pratesi G, Petrangolini G, Coradini D (2005). Isolation and in vitro propagation of tumorigenic breast cancer cells with stem/progenitor cell properties. Can Res.

[25] Zalpoor H, Bakhtiyari M, Akbari A, Aziziyan F, Shapourian H, Liaghat M (2022). Potential role of autophagy induced by FLT3-ITD and acid ceramidase in acute myeloid leukemia chemo-resistance: new insights. Cell Commun Signal.

[26] Haraguchi N, Inoue H, Tanaka F, Mimori K, Utsunomiya T, Sasaki A (2006). Cancer stem cells in human gastrointestinal cancers. Hum Cell.

[27] Ricci-Vitiani L, Lombardi DG, Pilozzi E, Biffoni M, Todaro M, Peschle C (2007). Identification and expansion of human colon-cancer-initiating cells. Nature.

[28] Lathia JD, Mack SC, Mulkearns-Hubert EE, Valentim CL, Rich JN (2015). Cancer stem cells in glioblastoma. Genes Dev.

[29] Hermann PC, Huber SL, Herrler T, Aicher A, Ellwart JW, Guba M (2007). Distinct populations of cancer stem cells determine tumor growth and metastatic activity in human pancreatic cancer. Cell Stem Cell.

[30] Wang J, Li ZH, White J, Zhang LB (2014). Lung cancer stem cells and implications for future therapeutics. Cell Biochem Biophys.

[31] Colak S, Medema JP (2014). Cancer stem cells–important players in tumor therapy resistance. FEBS J.

[32] Zalpoor H, Akbari A, Nayerain Jazi N, Liaghat M, Bakhtiyari M (2022). Possible role of autophagy induced by COVID-19 in cancer progression, chemo-resistance, and tumor recurrence. Infectious Agents Cancer.

[33] Giancotti FG (2013). Mechanisms governing metastatic dormancy and reactivation. Cell.

[34] Kim WT, Ryu CJ (2017). Cancer stem cell surface markers on normal stem cells. BMB Rep.

[35] Cho Y, Lee HW, Kang HG, Kim HY, Kim SJ, Chun KH (2015). Cleaved CD44 intracellular domain supports activation of stemness factors and promotes tumorigenesis of breast cancer. Oncotarget.

[36] Cui J, Li P, Liu X, Hu H, Wei W (2015). Abnormal expression of the Notch and Wnt/beta-catenin signaling pathways in stem-like ALDH(hi)CD44(+) cells correlates highly with Ki-67 expression in breast cancer. Oncol Lett.

[37] Ponta H, Sherman L, Herrlich PA (2003). CD44: from adhesion molecules to signalling regulators. Nat Rev Mol Cell Biol.

[38] Su J, Wu S, Wu H, Li L, Guo T (2016). CD44 is functionally crucial for driving lung cancer stem cells metastasis through Wnt/beta-catenin-FoxM1-Twist signaling. Mol Carcinog.

[39] Brugnoli F, Grassilli S, Al-Qassab Y, Capitani S, Bertagnolo V (2019). CD133 in breast cancer cells: more than a stem cell marker. J Oncol.

[40] Li Z (2013). CD133: a stem cell biomarker and beyond. Exp Hematol Oncol.

[41] Balzar M, Winter MJ, de Boer CJ, Litvinov SV (1999). The biology of the 17–1A antigen (Ep-CAM). J Mol Med.

[42] Trzpis M, McLaughlin PM, de Leij LM, Harmsen MC (2007). Epithelial cell adhesion molecule: more than a carcinoma marker and adhesion molecule. Am J Pathol.

[43] Walcher L, Kistenmacher AK, Suo H, Kitte R, Dluczek S, Strauss A (2020). Cancer Stem Cells-Origins and Biomarkers: Perspectives for Targeted Personalized Therapies. Front Immunol.

[44] Akbarzadeh M, Maroufi NF, Tazehkand AP, Akbarzadeh M, Bastani S, Safdari R, et al. Current approaches in identification and isolation of cancer stem cells. J Cell Physiol. 2019;234(9):14759–72. 10.1002/jcp.28271 30741412

[45] Toledo-Guzman ME, Hernandez MI, Gomez-Gallegos AA, Ortiz-Sanchez E (2019). ALDH as a Stem Cell Marker in Solid Tumors. Curr Stem Cell Res Ther.

[46] Ding XW, Wu JH, Jiang CP (2010). ABCG2: a potential marker of stem cells and novel target in stem cell and cancer therapy. Life Sci.

[47] Pattabiraman DR, Weinberg RA (2014). Tackling the cancer stem cells - what challenges do they pose?. Nat Rev Drug Discovery.

[48] Hadjimichael C, Chanoumidou K, Papadopoulou N, Arampatzi P, Papamatheakis J, Kretsovali A (2015). Common stemness regulators of embryonic and cancer stem cells. World J Stem Cells.

[49] Vlashi E, Pajonk F (2015). Cancer stem cells, cancer cell plasticity and radiation therapy. Semin Cancer Biol.

[50] Martinez-Outschoorn UE, Peiris-Pagés M, Pestell RG, Sotgia F, Lisanti MP (2017). Cancer metabolism: a therapeutic perspective. Nat Rev Clin Oncol.

[51] Menendez J, Joven J, Cufí S, Corominas-Faja B, Oliveras-Ferraros C, Cuyàs E (2013). The Warburg effect version 2.0: metabolic reprogramming of cancer stem cells. Cell Cycle..

[52] Jang Y-Y, Sharkis SJ (2007). A low level of reactive oxygen species selects for primitive hematopoietic stem cells that may reside in the low-oxygenic niche. Blood.

[53] Hensley CT, Faubert B, Yuan Q, Lev-Cohain N, Jin E, Kim J (2016). Metabolic heterogeneity in human lung tumors. Cell.

[54] Kamphorst JJ, Nofal M, Commisso C, Hackett SR, Lu W, Grabocka E (2015). Human pancreatic cancer tumors are nutrient poor and tumor cells actively scavenge extracellular protein. Can Res.

[55] Warburg O (1956). On the origin of cancer cells. Science.

[56] Warburg O, Wind F, Negelein E (1927). The Metabolism of tumors in the body. J Gen Physiol.

[57] Peiris-Pages M, Martinez-Outschoorn UE, Pestell RG, Sotgia F, Lisanti MP (2016). Cancer stem cell metabolism. Breast Cancer Res.

[58] Liu P, Liao J, Tang Z, Wu W, Yang J, Zeng Z (2014). Metabolic regulation of cancer cell side population by glucose through activation of the Akt pathway. Cell Death Differ.

[59] Palorini R, Votta G, Balestrieri C, Monestiroli A, Olivieri S, Vento R (2014). Energy metabolism characterization of a novel cancer stem cell-L ike Line 3 AB-OS. J Cell Biochem.

[60] Shen Y-A, Wang C-Y, Hsieh Y-T, Chen Y-J, Wei Y-H (2015). Metabolic reprogramming orchestrates cancer stem cell properties in nasopharyngeal carcinoma. Cell Cycle.

[61] Schieber MS, Chandel NS (2013). ROS links glucose metabolism to breast cancer stem cell and EMT phenotype. Cancer Cell.

[62] Penkert J, Ripperger T, Schieck M, Schlegelberger B, Steinemann D, Illig T (2016). On metabolic reprogramming and tumor biology: a comprehensive survey of metabolism in breast cancer. Oncotarget.

[63] Dong C, Yuan T, Wu Y, Wang Y, Fan TW, Miriyala S (2013). Loss of FBP1 by Snail-mediated repression provides metabolic advantages in basal-like breast cancer. Cancer Cell.

[64] Chen K-Y, Liu X, Bu P, Lin C-S, Rakhilin N, Locasale JW, et al., editors. A metabolic signature of colon cancer initiating cells. 2014 36th annual international conference of the IEEE Engineering in Medicine and Biology Society. 2014;26:4759–62. 10.1109/EMBC.2014.6944688 PMC4302416 25571056

[65] Ciavardelli D, Rossi C, Barcaroli D, Volpe S, Consalvo A, Zucchelli M (2014). Breast cancer stem cells rely on fermentative glycolysis and are sensitive to 2-deoxyglucose treatment. Cell Death Dis..

[66] Goidts V, Bageritz J, Puccio L, Nakata S, Zapatka M, Barbus S (2012). RNAi screening in glioma stem-like cells identifies PFKFB4 as a key molecule important for cancer cell survival. Oncogene.

[67] Wolf A, Agnihotri S, Micallef J, Mukherjee J, Sabha N, Cairns R (2011). Hexokinase 2 is a key mediator of aerobic glycolysis and promotes tumor growth in human glioblastoma multiforme. J Exp Med.

[68] Yu Y, Wang Y-y, Wang Y-q, Wang X, Liu Y-Y, Wang J-T (2016). Antiangiogenic therapy using endostatin increases the number of ALDH+ lung cancer stem cells by generating intratumor hypoxia. Sci Rep.

[69] Conley SJ, Gheordunescu E, Kakarala P, Newman B, Korkaya H, Heath AN (2012). Antiangiogenic agents increase breast cancer stem cells via the generation of tumor hypoxia. Proc Natl Acad Sci.

[70] Mahase S, Rattenni RN, Wesseling P, Leenders W, Baldotto C, Jain R (2017). Hypoxia-mediated mechanisms associated with antiangiogenic treatment resistance in glioblastomas. Am J Pathol.

[71] Marie SKN, Shinjo SMO (2011). Metabolism and brain cancer. Clinics.

[72] Chae YC, Kim JH (2018). Cancer stem cell metabolism: target for cancer therapy. BMB Rep.

[73] Palorini R. VG, Balestrieri C, Monestiroli A, Olivieri S, Vento R (2014). Energy metabolism characterization of a novel cancer stem cell-like line 3AB-OS. J. Cell Biochem.

[74] Anderson AS, Roberts PC, Frisard MI, Hulver MW, Schmelz EM (2014). Ovarian tumor-initiating cells display a flexible metabolism. Exp Cell Res.

[75] Song KKH, Han C, Zhang J, Dash S, Lim K, Wu T (2015). Active glycolytic metabolism in CD133(+) hepatocellular cancer stem cells: regulation by MIR-122. Oncotarget.

[76] Malchenko SSS, Boyineni J, Bi Y, Margaryan NV, Guda MR, Kostenko Y, Tomita T, Davuluri RV, Velpula K, Hendrix MJC, Soares MB (2018). Characterization of brain tumor initiating cells isolated from an animal model of CNS primitive neuroectodermal tumors. Oncotarget.

[77] Panopoulos ADYO, Ruiz S, Kida YS, Diep D, Tautenhahn R, Herrerías A, Batchelder EM, Plongthongkum N, Lutz M, Berggren WT, Zhang K, Evans RM, Siuzdak G, Izpisua Belmonte JC (2012). The metabolome of induced pluripotent stem cells reveals metabolic changes occurring in somatic cell reprogramming. Cell Res.

[78] Liberti MV, Locasale JW (2016). The Warburg effect: how does it benefit cancer cells?. Trends Biochem Sci.

[79] Porporato PE, Dhup S, Dadhich RK, Copetti T, Sonveaux P (2011). Anticancer targets in the glycolytic metabolism of tumors: a comprehensive review. Front Pharmacol.

[80] Vazquez A, Kamphorst JJ, Markert EK, Schug ZT, Tardito S, Gottlieb E (2016). Cancer metabolism at a glance. J Cell Sci.

[81] Kondoh HLM, Gil J, Wang J, Degan P, Peters G, Martinez D, Carnero A, Beach D (2005). Glycolytic enzymes can modulate cellular life span. Cancer Res.

[82] Alptekin A, Ye B, Ding HF (2017). Transcriptional regulation of stem cell and cancer stem cell metabolism. Current Stem Cell Rep.

[83] Deshmukh A, Arfuso F, Newsholme P, Dharmarajan A. Regulation of cancer stem cell metabolism by secreted frizzled-related protein 4 (sFRP4). Cancers. 2018;31;10(2):40. 10.3390/cancers10020040 PMC5836072 29385093

[84] Folmes CD, Nelson TJ, Martinez-Fernandez A, Arrell DK, Lindor JZ, Dzeja PP (2011). Somatic oxidative bioenergetics transitions into pluripotency-dependent glycolysis to facilitate nuclear reprogramming. Cell Metab.

[85] Gabay M, Li Y, Felsher DW. MYC activation is a hallmark of cancer initiation and maintenance. Cold Spring Harbor perspectives in medicine. 2014; 4(6):a014241. 10.1101/cshperspect.a014241 PMC4031954 24890832

[86] Gaude E, Frezza C (2016). Tissue-specific and convergent metabolic transformation of cancer correlates with metastatic potential and patient survival. Nat Commun.

[87] Zhou Y. ST, Feng L, Chen Z, Ogasawara M, Keating M. J (2011). Metabolic alterations in highly tumorigenic glioblastoma cells: preference for hypoxia and high dependency on glycolysis. JBiol Chem.

[88] Yuan SWF, Chen G, Zhang H, Feng L, Wang L (2013). Effective elimination of cancer stem cells by a novel drug combination strategy. Stem cells.

[89] Lee WT, St JJ (2015). The control of mitochondrial DNA replication during development and tumorigenesis. Ann N Y Acad Sci.

[90] Cairns RA, Harris IS, Mak TW (2011). Regulation of cancer cell metabolism. Nat Rev Cancer.

[91] Doherty JR, Yang C, Scott KE, Cameron MD, Fallahi M, Li W (2014). Blocking lactate export by inhibiting the Myc target MCT1 Disables glycolysis and glutathione synthesis. Can Res.

[92] Fischer K, Hoffmann P, Voelkl S, Meidenbauer N, Ammer J, Edinger M (2007). Inhibitory effect of tumor cell-derived lactic acid on human T cells. Blood.

[93] Pastò A, Bellio C, Pilotto G, Ciminale V, Silic-Benussi M, Guzzo G (2014). Cancer stem cells from epithelial ovarian cancer patients privilege oxidative phosphorylation, and resist glucose deprivation. Oncotarget.

[94] Sato M, Kawana K, Adachi K, Fujimoto A, Yoshida M, Nakamura H (2016). Spheroid cancer stem cells display reprogrammed metabolism and obtain energy by actively running the tricarboxylic acid (TCA) cycle. Oncotarget.

[95] Gao C, Shen Y, Jin F, Miao Y, Qiu X (2016). Cancer stem cells in small cell lung cancer cell line H446: higher dependency on oxidative phosphorylation and mitochondrial substrate-level phosphorylation than non-stem cancer cells. PLoS One.

[96] Vlashi E, Lagadec C, Vergnes L, Matsutani T, Masui K, Poulou M (2011). Metabolic state of glioma stem cells and nontumorigenic cells. Proc Natl Acad Sci.

[97] Janiszewska M, Suvà ML, Riggi N, Houtkooper RH, Auwerx J, Clément-Schatlo V (2012). Imp2 controls oxidative phosphorylation and is crucial for preserving glioblastoma cancer stem cells. Genes Dev.

[98] Viale A, Pettazzoni P, Lyssiotis CA, Ying H, Sánchez N, Marchesini M (2014). Oncogene ablation-resistant pancreatic cancer cells depend on mitochondrial function. Nature.

[99] LeBleu VS, O’Connell JT, Gonzalez Herrera KN, Wikman H, Pantel K, Haigis MC (2014). PGC-1α mediates mitochondrial biogenesis and oxidative phosphorylation in cancer cells to promote metastasis. Nat Cell Biol.

[100] Jiang WG, Douglas-Jones A, Mansel RE (2003). Expression of peroxisome-proliferator activated receptor-gamma (PPARγ) and the PPARγ co-activator, PGC-1, in human breast cancer correlates with clinical outcomes. Int J Cancer.

[101] De Luca A, Fiorillo M, Peiris-Pagès M, Ozsvari B, Smith DL, Sanchez-Alvarez R (2015). Mitochondrial biogenesis is required for the anchorage-independent survival and propagation of stem-like cancer cells. Oncotarget.

[102] Lamb R, Harrison H, Hulit J, Smith DL, Lisanti MP, Sotgia F (2014). Mitochondria as new therapeutic targets for eradicating cancer stem cells: Quantitative proteomics and functional validation via MCT1/2 inhibition. Oncotarget.

[103] Sancho P, Burgos-Ramos E, Tavera A, Kheir TB, Jagust P, Schoenhals M (2015). MYC/PGC-1α balance determines the metabolic phenotype and plasticity of pancreatic cancer stem cells. Cell Metab.

[104] Vlashi E, Lagadec C, Vergnes L, Reue K, Frohnen P, Chan M (2014). Metabolic differences in breast cancer stem cells and differentiated progeny. Breast Cancer Res Treat.

[105] Farnie G, Sotgia F, Lisanti MP (2015). High mitochondrial mass identifies a sub-population of stem-like cancer cells that are chemo-resistant. Oncotarget.

[106] Vazquez F, Lim J-H, Chim H, Bhalla K, Girnun G, Pierce K (2013). PGC1α expression defines a subset of human melanoma tumors with increased mitochondrial capacity and resistance to oxidative stress. Cancer Cell.

[107] Xie Q, Wu Q, Horbinski CM, Flavahan WA, Yang K, Zhou W (2015). Mitochondrial control by DRP1 in brain tumor initiating cells. Nat Neurosci.

[108] Katajisto P, Döhla J, Chaffer CL, Pentinmikko N, Marjanovic N, Iqbal S (2015). Asymmetric apportioning of aged mitochondria between daughter cells is required for stemness. Science.

[109] Prieto J, León M, Ponsoda X, Sendra R, Bort R, Ferrer-Lorente R (2016). Early ERK1/2 activation promotes DRP1-dependent mitochondrial fission necessary for cell reprogramming. Nat Commun.

[110] von Eyss B, Jaenicke LA, Kortlever RM, Royla N, Wiese KE, Letschert S (2015). A MYC-driven change in mitochondrial dynamics limits YAP/TAZ function in mammary epithelial cells and breast cancer. Cancer Cell.

[111] Guha M, Srinivasan S, Ruthel G, Kashina A, Carstens R, Mendoza A (2014). Mitochondrial retrograde signaling induces epithelial–mesenchymal transition and generates breast cancer stem cells. Oncogene.

[112] Ye XQ, Li Q, Wang GH, Sun FF, Huang GJ, Bian XW (2011). Mitochondrial and energy metabolism-related properties as novel indicators of lung cancer stem cells. Int J Cancer.

[113] Caria PTL, Dettori T, Murgia F, Santoru ML, Griffin JL, Vanni R, Atzori L (2018). Metabolomic alterations in thyrospheres and adherent parental cells in papillary thyroid carcinoma cell lines: a pilot study. Int J Mol Sci.

[114] Lagadinou ED, Sach A, Callahan K, Rossi RM, Neering SJ, Minhajuddin M (2013). BCL-2 inhibition targets oxidative phosphorylation and selectively eradicates quiescent human leukemia stem cells. Cell Stem Cell.

[115] Sato MKK, Adachi K, Fujimoto A, Yoshida M, Nakamura H, Nishida H, Inoue T, Taguchi A, Takahashi J, Eguchi S, Yamashita A, Tomio K, Wada-Hiraike O, Oda K, Nagamatsu T, Osuga Y, Fujii T (2016). Spheroid cancer stem cells display reprogrammed metabolism and obtain energy by actively running the tricarboxylic acid (TCA) cycle. Oncotarget.

[116] Lamb RFM, Chadwick A, Ozsvari B, Reeves KJ, Smith DL, Clarke RB, Howell SJ, Cappello AR, Martinez-Outschoorn UE, Peiris-Pagès M, Sotgia F, Lisanti MP (2015). Doxycycline down-regulates DNA-PK and radiosensitizes tumor initiating cells: Implications for more effective radiation therapy. Oncotarget.

[117] Yu LLM, Jia D, Ma J, Ben-Jacob E, Levine H, Kaipparettu BA, Onuchic JN (2017). Modeling the genetic regulation of cancer metabolism: interplay between glycolysis and oxidative phosphorylation. Cancer Res.

[118] Caino MCGJ, Chae YC, Vaira V, Rivadeneira DB, Faversani A, Rampini P, Kossenkov AV, Aird KM, Zhang R, Webster MR, Weeraratna AT, Bosari S, Languino LR, Altieri DC (2015). PI3K therapy reprograms mitochondrial trafficking to fuel tumor cell invasion. Proc Natl Acad Sci U S A.

[119] Ishikawa KTK, Akimoto M, Koshikawa N, Yamaguchi A, Imanishi H, Nakada K, Honma Y, Hayashi J (2008). ROS-generating mitochondrial DNA mutations can regulate tumor cell metastasis. Science.

[120] Farnie GSF, Lisanti MP (2015). High mitochondrial mass identifies a sub-population of stem-like cancer cells that are chemo-resistant. Oncotarget.

[121] De Luca AFM, Peiris-Pagès M, Ozsvari B, Smith DL, Sanchez-Alvarez R, Martinez-Outschoorn UE, Cappello AR, Pezzi V, Lisanti MP, Sotgia F (2015). Mitochondrial biogenesis is required for the anchorage-independent survival and propagation of stem-like cancer cells. Oncotarget.

[122] Wey AKP (2010). c-myc and N-myc promote active stem cell metabolism and cycling as architects of the developing brain. Oncotarget.

[123] Biasutto LDL, Zoratti M, Neuzil J (2010). Mitochondrially targeted anti-cancer agents. Mitochondrion.

[124] Jung JWPS, Lee SJ, Seo MS, Trosko JE, Kang KS (2011). Metformin represses self-renewal of the human breast carcinoma stem cells via inhibition of estrogen receptor-mediated OCT4 expression. PLoS One.

[125] Lamb ROB, Lisanti CL, Tanowitz HB, Howell A, Martinez-Outschoorn UE, Sotgia F, Lisanti MP (2015). Antibiotics that target mitochondria effectively eradicate cancer stem cells, across multiple tumor types: treating cancer like an infectious disease. Oncotarget.

[126] Cluntun AA, Lukey MJ, Cerione RA, Locasale JW (2017). Glutamine Metabolism in Cancer: Understanding the Heterogeneity. Trends Cancer.

[127] Kim JH, Lee KJ, Seo Y, Kwon JH, Yoon JP, Kang JY (2018). Effects of metformin on colorectal cancer stem cells depend on alterations in glutamine metabolism. Sci Rep.

[128] Oburoglu L, Tardito S, Fritz V, de Barros SC, Merida P, Craveiro M (2014). Glucose and glutamine metabolism regulate human hematopoietic stem cell lineage specification. Cell Stem Cell.

[129] Rysman E, Brusselmans K, Scheys K, Timmermans L, Derua R, Munck S (2010). De novo lipogenesis protects cancer cells from free radicals and chemotherapeutics by promoting membrane lipid saturation. Can Res.

[130] Staubach S, Hanisch FG (2011). Lipid rafts: signaling and sorting platforms of cells and their roles in cancer. Expert Rev Proteomics.

[131] Lai KKY, Kweon SM, Chi F, Hwang E, Kabe Y, Higashiyama R (2017). Stearoyl-CoA desaturase promotes liver fibrosis and tumor development in Mice via a Wnt positive-signaling loop by stabilization of low-density lipoprotein-receptor-related proteins 5 and 6. Gastroenterology.

[132] Liu X, Wu S, Yang Y, Zhao M, Zhu G, Hou Z (2017). The prognostic landscape of tumor-infiltrating immune cell and immunomodulators in lung cancer. Biomedicine Pharmacotherapy = Biomedecine Pharmacotherapie.

[133] Noto A, Raffa S, De Vitis C, Roscilli G, Malpicci D, Coluccia P (2013). Stearoyl-CoA desaturase-1 is a key factor for lung cancer-initiating cells. Cell Death Dis.

[134] Ali A, Levantini E, Teo JT, Goggi J, Clohessy JG, Wu CS, et al. Fatty acid synthase mediates EGFR palmitoylation in EGFR mutated non-small cell lung cancer. EMBO Mol Med. 2018;10(3):e8313. 10.15252/emmm.201708313 PMC5840543 29449326

[135] Chen C-L, Kumar DBU, Punj V, Xu J, Sher L, Tahara SM (2016). NANOG metabolically reprograms tumor-initiating stem-like cells through tumorigenic changes in oxidative phosphorylation and fatty acid metabolism. Cell Metab.

[136] De Francesco EM, Maggiolini M, Tanowitz HB, Sotgia F, Lisanti MP (2017). Targeting hypoxic cancer stem cells (CSCs) with Doxycycline: implications for optimizing anti-angiogenic therapy. Oncotarget.

[137] Tirinato L, Liberale C, Di Franco S, Candeloro P, Benfante A, La Rocca R (2015). Lipid droplets: a new player in colorectal cancer stem cells unveiled by spectroscopic imaging. Stem cells.

[138] Corominas-Faja B, Cuyàs E, Gumuzio J, Bosch-Barrera J, Leis O, Martin ÁG (2014). Chemical inhibition of acetyl-CoA carboxylase suppresses self-renewal growth of cancer stem cells. Oncotarget.

[139] Pandey PR, Okuda H, Watabe M, Pai SK, Liu W, Kobayashi A (2011). Resveratrol suppresses growth of cancer stem-like cells by inhibiting fatty acid synthase. Breast Cancer Res Treat.

[140] Yasumoto Y, Miyazaki H, Vaidyan LK, Kagawa Y, Ebrahimi M, Yamamoto Y (2016). Inhibition of fatty acid synthase decreases expression of stemness markers in glioma stem cells. PLoS One.

[141] Kathagen A, Schulte A, Balcke G, Phillips HS, Martens T, Matschke J (2013). Hypoxia and oxygenation induce a metabolic switch between pentose phosphate pathway and glycolysis in glioma stem-like cells. Acta Neuropathol.

[142] Li D, Fu Z, Chen R, Zhao X, Zhou Y, Zeng B (2015). Inhibition of glutamine metabolism counteracts pancreatic cancer stem cell features and sensitizes cells to radiotherapy. Oncotarget.

[143] Lu W, Pelicano H, Huang P (2010). Cancer metabolism: is glutamine sweeter than glucose?. Cancer Cell.

[144] Shelton LM, Huysentruyt LC, Seyfried TN (2010). Glutamine targeting inhibits systemic metastasis in the VM-M3 murine tumor model. Int J Cancer.

[145] Li H, Guo L, Li J-W, Liu N, Qi R, Liu J (2000). Expression of hyaluronan receptors CD44 and RHAMM in stomach cancers: relevance with tumor progression. Int J Oncol.

[146] Dosio F, Arpicco S, Stella B, Fattal E (2016). Hyaluronic acid for anticancer drug and nucleic acid delivery. Adv Drug Deliv Rev.

[147] Saeg F, Anbalagan M. Breast cancer stem cells and the challenges of eradication: a review of novel therapies. Stem cell investigation. 2018;5. 10.21037/sci.2018.10.05 PMC6232051 30498750

[148] Günthert U, Hofmann M, Rudy W, Reber S, Zöller M, Hauβmann I (1991). A new variant of glycoprotein CD44 confers metastatic potential to rat carcinoma cells. Cell.

[149] Wang L, Zuo X, Xie K, Wei D. The role of CD44 and cancer stem cells. Cancer stem cells: methods and protocols. New York: Springer New York; 2018. p. 31–42. 10.1007/978-1-4939-7401-6_3 28986884

[150] Wu R-L, Huang L, Zhao H-C, Geng X-P (2017). Hyaluronic acid in digestive cancers. J Cancer Res Clin Oncol.

[151] Abatangelo G, Vindigni V, Avruscio G, Pandis L, Brun P (2020). Hyaluronic acid: redefining its role. Cells.

[152] Mima K, Beppu T, Ishiko T, Chikamoto A, Nakagawa S, Hayashi H (2014). Preoperative serum hyaluronic acid level as a prognostic factor in patients undergoing hepatic resection for hepatocellular carcinoma. J Br Surg.

[153] Liu Q, Luo Q, Ju Y, Song G (2020). Role of the mechanical microenvironment in cancer development and progression. Cancer Biol Med.

[154] Tian BR, Lin WF, Zhang Y (2021). Effects of biomechanical forces on the biological behavior of cancer stem cells. J Cancer.

[155] Nallanthighal S, Heiserman JP, Cheon D-J (2019). The role of the extracellular matrix in cancer stemness. Front Cell Dev Biol.

[156] You Y, Zheng Q, Dong Y, Xie X, Wang Y, Wu S (2016). Matrix stiffness-mediated effects on stemness characteristics occurring in HCC cells. Oncotarget.

[157] Jokela TA, LaBarge MA (2021). Integration of mechanical and ECM microenvironment signals in the determination of cancer stem cell states. Curr Stem Cell Rep.

[158] Chapeland-Leclerc F, Dilmaghani A, Ez-Zaki L, Boisnard S, Da Silva B, Gaslonde T (2015). Systematic gene deletion and functional characterization of histidine kinase phosphorelay receptors (HKRs) in the human pathogenic fungus Aspergillus fumigatus. Fungal Genet Biol.

[159] Jiang Y, Zhang H, Wang J, Liu Y, Luo T, Hua H (2022). Targeting extracellular matrix stiffness and mechanotransducers to improve cancer therapy. J Hematol Oncol.

[160] Tian B, Luo Q, Ju Y, Song G (2019). A soft matrix enhances the cancer stem cell phenotype of HCC cells. Int J Mol Sci.

[161] Triantafillu UL, Park S, Klaassen NL, Raddatz AD, Kim Y (2017). Fluid shear stress induces cancer stem cell-like phenotype in MCF7 breast cancer cell line without inducing epithelial to mesenchymal transition. Int J Oncol.

[162] Kalluri R, Weinberg RA (2009). The basics of epithelial-mesenchymal transition. J Clin Investig.

[163] Lamouille S, Xu J, Derynck R (2014). Molecular mechanisms of epithelial-mesenchymal transition. Nat Rev Mol Cell Biol.

[164] Du B, Shim JS. Targeting Epithelial-Mesenchymal Transition (EMT) to overcome drug resistance in cancer. Molecules. 2016;21(7):965. 10.3390/molecules21070965 PMC6273543 27455225

[165] Liu X, Yun F, Shi L, Li ZH, Luo NR, Jia YF (2015). Roles of signaling pathways in the epithelial-mesenchymal transition in cancer. Asian Pac J Cancer Prev.

[166] Nieto MA, Huang RY, Jackson RA, Thiery JP (2016). Emt: 2016. Cell.

[167] Mani SA, Guo W, Liao MJ, Eaton EN, Ayyanan A, Zhou AY (2008). The epithelial-mesenchymal transition generates cells with properties of stem cells. Cell.

[168] Polyak K, Weinberg RA (2009). Transitions between epithelial and mesenchymal states: acquisition of malignant and stem cell traits. Nat Rev Cancer.

[169] Topcul MCI (2016). Clinical significance of epithelial-mesenchymal transition and cancer stem cells. J BUON.

[170] Wang R, Sun Q, Wang P, Liu M, Xiong S, Luo J (2016). Notch and Wnt/beta-catenin signaling pathway play important roles in activating liver cancer stem cells. Oncotarget.

[171] Lee SY, Jeon HM, Ju MK, Jeong EK, Kim CH, Yoo MA (2015). Dlx-2 is implicated in TGF-beta- and Wnt-induced epithelial-mesenchymal, glycolytic switch, and mitochondrial repression by Snail activation. Int J Oncol.

[172] Lu J, Tan M, Cai Q (2015). The Warburg effect in tumor progression: mitochondrial oxidative metabolism as an anti-metastasis mechanism. Cancer letters..

[173] Denko NC (2008). Hypoxia, HIF1 and glucose metabolism in the solid tumour. Nat Rev Cancer.

[174] Lee SY, Jeon HM, Ju MK, Kim CH, Yoon G, Han SI (2012). Wnt/Snail signaling regulates cytochrome C oxidase and glucose metabolism. Can Res.

[175] Pate KT, Stringari C, Sprowl-Tanio S, Wang K, TeSlaa T, Hoverter NP (2014). Wnt signaling directs a metabolic program of glycolysis and angiogenesis in colon cancer. EMBO J.

[176] Thompson CB (2014). Wnt meets Warburg: another piece in the puzzle?. EMBO J.

[177] Kondaveeti Y, Guttilla Reed IK, White BA (2015). Epithelial-mesenchymal transition induces similar metabolic alterations in two independent breast cancer cell lines. Cancer Lett.

[178] I. Akalay BJ, Hasmim M., Noman M.Z., Andre F., De Cremoux P. (2013). Epithelial-to-mesenchymal transition and autophagy induction in breast carcinoma promote escape from T-cell-mediated lysis. Cancer Res.

[179] Meijer TW, Kaanders JH, Span PN, Bussink J (2012). Targeting hypoxia, HIF-1, and tumor glucose metabolism to improve radiotherapy efficacy. Clin Cancer Res.

[180] Cabrera MC, Hollingsworth RE, Hurt EM (2015). Cancer stem cell plasticity and tumor hierarchy. World J Stem Cells.

[181] Zeuner A, Todaro M, Stassi G, De Maria R (2014). Colorectal cancer stem cells: from the crypt to the clinic. Cell Stem Cell.

[182] Ahmed F, Haass NK (2018). Microenvironment-driven dynamic heterogeneity and phenotypic plasticity as a mechanism of melanoma therapy resistance. Front Oncol.

[183] Davies AE, Albeck JG (2018). Microenvironmental Signals and Biochemical Information Processing: Cooperative Determinants of Intratumoral Plasticity and Heterogeneity. Front Cell Dev Biol.

[184] Luo M, Shang L, Brooks MD, Jiagge E, Zhu Y, Buschhaus JM (2018). Targeting Breast Cancer Stem Cell State Equilibrium through Modulation of Redox Signaling. Cell Metab..

[185] Badve SNH (2012). Breast-cancer stem cells-beyond semantics. Lancet Oncol.

[186] Das PK, Pillai S, Rakib MA, Khanam JA, Gopalan V, Lam AKY (2020). Plasticity of cancer stem cell: origin and role in disease progression and therapy resistance. Stem Cell Rev Rep.

[187] Lathia JD, Heddleston JM, Venere M, Rich JN (2011). Deadly teamwork: neural cancer stem cells and the tumor microenvironment. Cell Stem Cell.

[188] Comoglio PM, Trusolino L, Boccaccio C (2018). Known and novel roles of the MET oncogene in cancer: a coherent approach to targeted therapy. Nat Rev Cancer.

[189] Medema JP, Vermeulen L (2011). Microenvironmental regulation of stem cells in intestinal homeostasis and cancer. Nature.

[190] Schwitalla S, Fingerle AA, Cammareri P, Nebelsiek T, Goktuna SI, Ziegler PK (2013). Intestinal tumorigenesis initiated by dedifferentiation and acquisition of stem-cell-like properties. Cell.

[191] Tang YA, Chen YF, Bao Y, Mahara S, Yatim S, Oguz G (2018). Hypoxic tumor microenvironment activates GLI2 via HIF-1alpha and TGF-beta2 to promote chemoresistance in colorectal cancer. Proc Natl Acad Sci USA.

[192] Lugano R, Ramachandran M, Dimberg A (2020). Tumor angiogenesis: causes, consequences, challenges and opportunities. Cell Mol Life Sci.

[193] Oswald J, Boxberger S, Jorgensen B, Feldmann S, Ehninger G, Bornhauser M (2004). Mesenchymal stem cells can be differentiated into endothelial cells in vitro. Stem cells.

[194] Song YS, Lee HJ, Park IH, Kim WK, Ku JH, Kim SU (2007). Potential differentiation of human mesenchymal stem cell transplanted in rat corpus cavernosum toward endothelial or smooth muscle cells. Int J Impot Res.

[195] Beckermann BM, Kallifatidis G, Groth A, Frommhold D, Apel A, Mattern J (2008). VEGF expression by mesenchymal stem cells contributes to angiogenesis in pancreatic carcinoma. Br J Cancer.

[196] Liu TJ, Sun BC, Zhao XL, Zhao XM, Sun T, Gu Q (2013). CD133+ cells with cancer stem cell characteristics associates with vasculogenic mimicry in triple-negative breast cancer. Oncogene.

[197] Wang X, Cao Y, Zhang S, Chen Z, Fan L, Shen X (2017). Stem cell autocrine CXCL12/CXCR4 stimulates invasion and metastasis of esophageal cancer. Oncotarget.

[198] Ping YF, Yao XH, Jiang JY, Zhao LT, Yu SC, Jiang T (2011). The chemokine CXCL12 and its receptor CXCR4 promote glioma stem cell-mediated VEGF production and tumour angiogenesis via PI3K/AKT signalling. J Pathol.

[199] Frank NY, Schatton T, Kim S, Zhan Q, Wilson BJ, Ma J (2011). VEGFR-1 expressed by malignant melanoma-initiating cells is required for tumor growth. Can Res.

[200] Wang SS, Gao XL, Liu X, Gao SY, Fan YL, Jiang YP (2016). CD133+ cancer stem-like cells promote migration and invasion of salivary adenoid cystic carcinoma by inducing vasculogenic mimicry formation. Oncotarget.

[201] Wang R, Chadalavada K, Wilshire J, Kowalik U, Hovinga KE, Geber A (2010). Glioblastoma stem-like cells give rise to tumour endothelium. Nature.

[202] Te Boekhorst V, Friedl P (2016). Plasticity of cancer cell invasion-mechanisms and implications for therapy. Adv Cancer Res.

[203] Nandy SB, Lakshmanaswamy R (2017). Cancer stem cells and metastasis. Prog Mol Biol Transl Sci.

[204] Bai X, Li YY, Zhang HY, Wang F, He HL, Yao JC (2017). Role of matrix metalloproteinase-9 in transforming growth factor-beta1-induced epithelial-mesenchymal transition in esophageal squamous cell carcinoma. Onco Targets Ther.

[205] Ren Y, Jia HH, Xu YQ, Zhou X, Zhao XH, Wang YF (2018). Paracrine and epigenetic control of CAF-induced metastasis: the role of HOTAIR stimulated by TGF-ss1 secretion. Mol Cancer.

[206] Ye XZ, Xu SL, Xin YH, Yu SC, Ping YF, Chen L (2012). Tumor-associated microglia/macrophages enhance the invasion of glioma stem-like cells via TGF-beta1 signaling pathway. J Immunol.

[207] Hiratsuka S, Watanabe A, Aburatani H, Maru Y (2006). Tumour-mediated upregulation of chemoattractants and recruitment of myeloid cells predetermines lung metastasis. Nat Cell Biol.

[208] Kaplan RN, Riba RD, Zacharoulis S, Bramley AH, Vincent L, Costa C (2005). VEGFR1-positive haematopoietic bone marrow progenitors initiate the pre-metastatic niche. Nature.

[209] Li R, Yuan B, Zhang Y, Dai J, Zhang P, Fang F (2016). Vascular endothelial growth factor secreted by breast cancer cells plays a critical role in the formation of pre-metastatic niche in the mouse lung. Zhonghua Zhong Liu Za Zhi.

[210] Yan HH, Jiang J, Pang Y, Achyut BR, Lizardo M, Liang X (2015). CCL9 Induced by TGFbeta Signaling in Myeloid Cells Enhances Tumor Cell Survival in the Premetastatic Organ. Can Res.

[211] Cojoc M, Peitzsch C, Trautmann F, Polishchuk L, Telegeev GD, Dubrovska A (2013). Emerging targets in cancer management: role of the CXCL12/CXCR4 axis. Onco Targets Ther.

[212] Roato IFR (2018). Cancer stem cells, bone and tumor microenvironment: key players in bone metastases. Cancers.

[213] Kato Y, Ozawa S, Tsukuda M, Kubota E, Miyazaki K, St-Pierre Y (2007). Acidic extracellular pH increases calcium influx-triggered phospholipase D activity along with acidic sphingomyelinase activation to induce matrix metalloproteinase-9 expression in mouse metastatic melanoma. FEBS J.

[214] Kindzelskii AL, Amhad I, Keller D, Zhou MJ, Haugland RP, Garni-Wagner BA (2004). Pericellular proteolysis by leukocytes and tumor cells on substrates: focal activation and the role of urokinase-type plasminogen activator. Histochem Cell Biol.

[215] Mohamed MM, Sloane BF (2006). Cysteine cathepsins: multifunctional enzymes in cancer. Nat Rev Cancer.

[216] Gatenby RA, Gawlinski ET, Gmitro AF, Kaylor B, Gillies RJ (2006). Acid-mediated tumor invasion: a multidisciplinary study. Can Res.

[217] Araki K, Shimura T, Yajima T, Tsutsumi S, Suzuki H, Okada K (2009). Phosphoglucose isomerase/autocrine motility factor promotes melanoma cell migration through ERK activation dependent on autocrine production of interleukin-8. J Biol Chem.

[218] Shimizu KTM, Watanabe H, Nagamachi Y, Niinaka Y, Shiroishi T, Ohwada S, Raz A, Yokota J (1999). The autocrine motility factor receptor gene encodes a novel type of seven transmembrane protein. FEBS Lett.

[219] Thiery JP (2002). Epithelial-mesenchymal transitions in tumour progression. Nat Rev Cancer.

[220] Wang JMTG, Matsushima K, Van Damme J, Mantovani A (1990). Induction of haptotactic migration of melanoma cells by neutrophil activating protein/interleukin-8. Biochem Biophys Res Commun.

[221] Zhu XY, Wang L, Luan SH, Zhang HS, Huang WT, Wang NH (2014). The PGI-KLF4 pathway regulates self-renewal of glioma stem cells residing in the mesenchymal niches in human gliomas. Neoplasma.

[222] Wang LY, Liu YP, Chen LG, Chen YL, Tan L, Liu JJ (2013). Pyruvate kinase M2 plays a dual role on regulation of the EGF/EGFR signaling via E-cadherin-dependent manner in gastric cancer cells. PLoS One.

[223] Zhou Z, Li M, Zhang L, Zhao H, Sahin O, Chen J (2018). Oncogenic Kinase-induced PKM2 tyrosine 105 phosphorylation converts Nononcogenic PKM2 to a tumor promoter and induces cancer stem-like cells. Can Res.

[224] Baumann F, Leukel P, Doerfelt A, Beier CP, Dettmer K, Oefner PJ (2009). Lactate promotes glioma migration by TGF-beta2-dependent regulation of matrix metalloproteinase-2. Neuro Oncol.

[225] Cui B, Luo Y, Tian P, Peng F, Lu J, Yang Y (2019). Stress-induced epinephrine enhances lactate dehydrogenase A and promotes breast cancer stem-like cells. J Clin Investig.

[226] Hanahan D, Coussens LM (2012). Accessories to the crime: functions of cells recruited to the tumor microenvironment. Cancer Cell.

[227] Eble JANS (2019). The extracellular matrix in tumor progression and metastasis. Clin Exp Metastasis.

[228] Walker CME, Del Río HA (2018). Role of extracellular matrix in development and cancer progression. Int J Mol Sci.

[229] Samanta DGD, Chaturvedia P, Xiang L, Semenza GL (2014). Hypoxia-inducible factors are required for chemotherapy resistance of breast cancer stem cells. Proc Natl Acad Sci U S A.

[230] Henke ENR, Ergün S (2020). 2020;6: Extracellular matrix in the tumor microenvironment and its impact on cancer therapy. Front Mol Biosci.

[231] Wu TDY (2017). Tumor microenvironment and therapeutic response. Cancer Lett.

[232] Hui L, Chen Y (2015). Tumor microenvironment: Sanctuary of the devil. Cancer Lett.

[233] Crusz SM, Balkwill FR (2015). Inflammation and cancer: advances and new agents. Nat Rev Clin Oncol.

[234] Grivennikov SI, Greten FR, Karin M (2010). Immunity, inflammation, and cancer. Cell.

[235] Jarnicki A, Putoczki T, Ernst M (2010). Stat3: linking inflammation to epithelial cancer - more than a "gut" feeling?. Cell Div.

[236] Colotta F, Allavena P, Sica A, Garlanda C, Mantovani A (2009). Cancer-related inflammation, the seventh hallmark of cancer: links to genetic instability. Carcinogenesis.

[237] Chen X, Xu H, Yuan P, Fang F, Huss M, Vega VB (2008). Integration of external signaling pathways with the core transcriptional network in embryonic stem cells. Cell.

[238] Dethlefsen C, Hojfeldt G, Hojman P (2013). The role of intratumoral and systemic IL-6 in breast cancer. Breast Cancer Res Treat.

[239] Prieto-Vila MTR, Usuba W, Kohama I, Ochiya T (2017). Drug resistance driven by cancer stem cells and their niche. Int J Mol Sci.

[240] Ekström EJBC, von Bülow V, Serifler F, Carlemalm E, Jönsson G (2014). WNT5A induces release of exosomes containing pro-angiogenic and immunosuppressive factors from malignant melanoma cells. Mol Cancer.

[241] Skog JWT, van Rijn S, Meijer DH, Gainche L, Curry WT (2008). Glioblastoma microvesicles transport RNA and proteins that promote tumour growth and provide diagnostic biomarkers. Nat Cell Biol.

[242] Taraboletti GDAS, Giusti I, Marchetti D, Borsotti P, Millimaggi D (2006). Bioavailability of VEGF in tumor-shed vesicles depends on vesicle burst induced by acidic pH 1. Neoplasia.

[243] Chen XLH, Zhang J, Zen K, Zhang CY (2012). Secreted microRNAs: a new form of intercellular communication. Trends Cell Biol.

[244] Webber JSR, Mason MD, Tabi Z, Clayton A (2010). Cancer exosomes trigger fibroblast to myofibroblast differentiation. Can Res.

[245] Kim JKT, Lee MS, Mun JY, Ihm C, Kim SA (2016). Exosome cargo reflects TGF-β1-mediated epithelial-to-mesenchymal transition (EMT) status in A549 human lung adenocarcinoma cells. Biochem Biophys Res Commun.

[246] Ye JWD, Wu P, Chen Z, Huang J (2014). The cancer stemcell niche: cross talk between cancer stemcells and their microenvironment. Tumor Biol.

[247] Snyder V, Reed-Newman TC, Arnold L, Thomas SM, Anant S (2018). Cancer stem cell metabolism and potential therapeutic targets. Front Oncol.

[248] Al-Zoughbi WHJ, Paramasivan GS, Till H, Pichler M, Guertl-Lackner B (2014). Tumor macroenvironment and metabolism. Semin Oncol.

[249] Mauer JDJ, Bruning JC (2015). Versatile functions for IL-6 in metabolism and cancer. Trends Immunol.

[250] Martinez-Outschoorn UE W-MD, Lin Z, Flomenberg N, Howell A, Pestell RG (2011). Cytokine production and inflammation drive autophagy in the tumor microenvironment: role of stromal caveolin-1 as a key regulator. Cell Cycle.

[251] Korkaya HLS, Wicha MS (2011). Breast cancer stem cells, cytokine networks, and the tumor microenvironment. J Clin Invest.

[252] Chiavarina B W-MD, Migneco G, Martinez-Outschoorn UE, Pavlides S, Howell A (2010). HIF1-alpha functions as a tumor promoter in cancer associated fibroblasts, and as a tumor suppressor in breast cancer cells: autophagy drives compartment-specific oncogenesis. Cell Cycle..

[253] Chiavarina B M-OU, Whitaker-Menezes D, Howell A, Tanowitz HB, Pestell RG (2012). Metabolic reprogramming and two-compartment tumor metabolism: opposing role(s) of HIF1alpha and HIF2alpha in tumor-associated fibroblasts and human breast cancer cells. Cell Cycle..

[254] Guido C W-MD, Capparelli C, Balliet R, Lin Z, Pestell RG (2012). Metabolic reprogramming of cancer-associated fibroblasts by TGF-beta drives tumor growth: connecting TGF-beta signaling with ‘Warburg-like’ cancer metabolism and L-lactate production. Cell Cycle..

[255] Martinez-Outschoorn UEPM, Ertel A, Tsirigos A, Lin Z, Pavlides S (2011). Ketones and lactate increase cancer cell ‘stemness’, driving recurrence, metastasis and poor clinical outcome in breast cancer: achieving personalized medicine via metabolo-genomics. Cell Cycle.

[256] Semenza GL (2014). Oxygen sensing, hypoxia-inducible factors, and disease pathophysiology. Annu Rev Pathol.

[257] ManoochehriKhoshinani H, Afshar S, Najafi R (2016). Hypoxia: a double-edged sword in cancer therapy. Cancer Invest.

[258] Ullmann P, Nurmik M, Begaj R, Haan S, Letellier E. Hypoxia- and microRNA-induced metabolic reprogramming of tumor-initiating cells. Cells. 2019;8(6):528. 10.3390/cells8060528 PMC6627778 31159361

[259] Riera-Domingo C, Audige A, Granja S, Cheng WC, Ho PC, Baltazar F (2020). Immunity, hypoxia, and metabolism-the Menage a trois of cancer: implications for immunotherapy. Physiol Rev.

[260] Flavahan WA, Wu Q, Hitomi M, Rahim N, Kim Y, Sloan AE (2013). Brain tumor initiating cells adapt to restricted nutrition through preferential glucose uptake. Nat Neurosci.

[261] Gordon N, Skinner AM, Pommier RF, Schillace RV, O'Neill S, Peckham JL (2015). Gene expression signatures of breast cancer stem and progenitor cells do not exhibit features of Warburg metabolism. Stem Cell Res Ther.

[262] Conley SJ, Gheordunescu E, Kakarala P, Newman B, Korkaya H, Heath AN (2012). Antiangiogenic agents increase breast cancer stem cells via the generation of tumor hypoxia. Proc Natl Acad Sci USA.

[263] Zalpoor H, Aziziyan F, Liaghat M, Bakhtiyari M, Akbari A, Nabi-Afjadi M (2022). The roles of metabolic profiles and intracellular signaling pathways of tumor microenvironment cells in angiogenesis of solid tumors. Cell Commun Signal.

[264] Conley SJ, Baker TL, Burnett JP, Theisen RL, Lazarus D, Peters CG (2015). CRLX101, an investigational camptothecin-containing nanoparticle-drug conjugate, targets cancer stem cells and impedes resistance to antiangiogenic therapy in mouse models of breast cancer. Breast Cancer Res Treat.

[265] Hezari S, Olad A, Dilmaghani A (2022). Modified gelatin/iron-based metal-organic framework nanocomposite hydrogel as wound dressing: Synthesis, antibacterial activity, and Camellia sinensis release. Int J Biol Macromol.

[266] Mansouri E, Tarhriz V, Yousefi V, Dilmaghani A (2020). Intercalation and release of an anti-inflammatory drug into designed three-dimensionally layered double hydroxide nanostructure via calcination–reconstruction route. Adsorption.

[267] Murakami A, Takahashi F, Nurwidya F, Kobayashi I, Minakata K, Hashimoto M (2014). Hypoxia increases gefitinib-resistant lung cancer stem cells through the activation of insulin-like growth factor 1 receptor. PLoS One.

[268] Li Z, Bao S, Wu Q, Wang H, Eyler C, Sathornsumetee S (2009). Hypoxia-inducible factors regulate tumorigenic capacity of glioma stem cells. Cancer Cell.

[269] Shi QY, Zhang SJ, Liu L, Chen QS, Yu LN, Zhang FJ (2015). Sevoflurane promotes the expansion of glioma stem cells through activation of hypoxia-inducible factors in vitro. Br J Anaesth.

[270] Deynoux M, Sunter N, Herault O, Mazurier F (2016). Hypoxia and Hypoxia-Inducible Factors in Leukemias. Front Oncol.

[271] Thomas S, Harding MA, Smith SC, Overdevest JB, Nitz MD, Frierson HF (2012). CD24 is an effector of HIF-1-driven primary tumor growth and metastasis. Can Res.

[272] Kalluri R (2016). The biology and function of fibroblasts in cancer. Nat Rev Cancer.

[273] Xing F, Saidou J, Watabe K (2010). Cancer associated fibroblasts (CAFs) in tumor microenvironment. Front Biosci.

[274] Jing Y, Han Z, Zhang S, Liu Y, Wei L (2011). Epithelial-Mesenchymal Transition in tumor microenvironment. Cell Biosci.

[275] Liao Z, Tan ZW, Zhu P, Tan NS (2019). Cancer-associated fibroblasts in tumor microenvironment - Accomplices in tumor malignancy. Cell Immunol.

[276] Donnenberg VS, Donnenberg AD, Zimmerlin L, Landreneau RJ, Bhargava R, Wetzel RA (2010). Localization of CD44 and CD90 positive cells to the invasive front of breast tumors. Cytometry B Clin Cytom.

[277] Liu FL, Mo EP, Yang L, Du J, Wang HS, Zhang H (2016). Autophagy is involved in TGF-beta1-induced protective mechanisms and formation of cancer-associated fibroblasts phenotype in tumor microenvironment. Oncotarget.

[278] Martinez-Outschoorn UE, Trimmer C, Lin Z, Whitaker-Menezes D, Chiavarina B, Zhou J (2010). Autophagy in cancer associated fibroblasts promotes tumor cell survival: role of hypoxia, HIF1 induction and NFkappaB activation in the tumor stromal microenvironment. Cell Cycle.

[279] Wang Q, Xue L, Zhang X, Bu S, Zhu X, Lai D (2016). Autophagy protects ovarian cancer-associated fibroblasts against oxidative stress. Cell Cycle.

[280] Sousa CM, Biancur DE, Wang X, Halbrook CJ, Sherman MH, Zhang L (2016). Pancreatic stellate cells support tumour metabolism through autophagic alanine secretion. Nature.

[281] Korkaya H, Liu S, Wicha MS (2011). Regulation of cancer stem cells by cytokine networks: attacking cancer's inflammatory roots. Clin Cancer Res.

[282] Yang L, Achreja A, Yeung TL, Mangala LS, Jiang D, Han C (2016). Targeting stromal glutamine synthetase in tumors disrupts tumor microenvironment-regulated cancer cell growth. Cell Metab.

[283] Chaudhri VK, Salzler GG, Dick SA, Buckman MS, Sordella R, Karoly ED (2013). Metabolic alterations in lung cancer-associated fibroblasts correlated with increased glycolytic metabolism of the tumor. Mol Cancer Res.

[284] Lisanti MP, Martinez-Outschoorn UE, Sotgia F (2013). Oncogenes induce the cancer-associated fibroblast phenotype: metabolic symbiosis and "fibroblast addiction" are new therapeutic targets for drug discovery. Cell Cycle.

[285] Sansone P, Savini C, Kurelac I, Chang Q, Amato LB, Strillacci A (2017). Packaging and transfer of mitochondrial DNA via exosomes regulate escape from dormancy in hormonal therapy-resistant breast cancer. Proc Natl Acad Sci USA.

[286] Pavlides S, Whitaker-Menezes D, Castello-Cros R, Flomenberg N, Witkiewicz AK, Frank PG (2009). The reverse Warburg effect: aerobic glycolysis in cancer associated fibroblasts and the tumor stroma. Cell Cycle.

[287] Bao S, Wu Q, Sathornsumetee S, Hao Y, Li Z, Hjelmeland AB (2006). Stem cell-like glioma cells promote tumor angiogenesis through vascular endothelial growth factor. Can Res.

[288] Folkins C, Shaked Y, Man S, Tang T, Lee CR, Zhu Z (2009). Glioma tumor stem-like cells promote tumor angiogenesis and vasculogenesis via vascular endothelial growth factor and stromal-derived factor 1. Can Res.

[289] Ricci-Vitiani L, Pallini R, Biffoni M, Todaro M, Invernici G, Cenci T (2010). Tumour vascularization via endothelial differentiation of glioblastoma stem-like cells. Nature.

[290] Charles N, Ozawa T, Squatrito M, Bleau AM, Brennan CW, Hambardzumyan D (2010). Perivascular nitric oxide activates notch signaling and promotes stem-like character in PDGF-induced glioma cells. Cell Stem Cell.

[291] Lu J, Ye X, Fan F, Xia L, Bhattacharya R, Bellister S (2013). Endothelial cells promote the colorectal cancer stem cell phenotype through a soluble form of Jagged-1. Cancer Cell.

[292] Tang DG (2012). Understanding cancer stem cell heterogeneity and plasticity. Cell Res.

[293] De Bock K, Georgiadou M, Schoors S, Kuchnio A, Wong BW, Cantelmo AR (2013). Role of PFKFB3-driven glycolysis in vessel sprouting. Cell.

[294] Polet F, Feron O (2013). Endothelial cell metabolism and tumour angiogenesis: glucose and glutamine as essential fuels and lactate as the driving force. J Intern Med.

[295] Verdegem D, Moens S, Stapor P, Carmeliet P (2014). Endothelial cell metabolism: parallels and divergences with cancer cell metabolism. Cancer Metab.

[296] Arany Z, Foo SY, Ma Y, Ruas JL, Bommi-Reddy A, Girnun G (2008). HIF-independent regulation of VEGF and angiogenesis by the transcriptional coactivator PGC-1alpha. Nature.

[297] Wright GL, Maroulakou IG, Eldridge J, Liby TL, Sridharan V, Tsichlis PN (2008). VEGF stimulation of mitochondrial biogenesis: requirement of AKT3 kinase. FASEB J.

[298] Lim AR, Rathmell WK, Rathmell JC. The tumor microenvironment as a metabolic barrier to effector T cells and immunotherapy. Elife. 2020;9:e55185. 10.7554/eLife.55185 PMC7200151 32367803

[299] Romero-Garcia S, Moreno-Altamirano MM, Prado-Garcia H, Sanchez-Garcia FJ (2016). Lactate contribution to the tumor microenvironment: mechanisms, effects on immune cells and therapeutic relevance. Front Immunol.

[300] Marchiq I, Le Floch R, Roux D, Simon MP, Pouyssegur J (2015). Genetic disruption of lactate/H+ symporters (MCTs) and their subunit CD147/BASIGIN sensitizes glycolytic tumor cells to phenformin. Can Res.

[301] Counillon L, Bouret Y, Marchiq I, Pouyssegur J (2016). Na(+)/H(+) antiporter (NHE1) and lactate/H(+) symporters (MCTs) in pH homeostasis and cancer metabolism. Biochim Biophys Acta.

[302] Binnewies M, Roberts EW, Kersten K, Chan V, Fearon DF, Merad M (2018). Understanding the tumor immune microenvironment (TIME) for effective therapy. Nat Med.

[303] Husain Z, Huang Y, Seth P, Sukhatme VP (2013). Tumor-derived lactate modifies antitumor immune response: effect on myeloid-derived suppressor cells and NK cells. J Immunol.

[304] Gottfried E, Kunz-Schughart LA, Ebner S, Mueller-Klieser W, Hoves S, Andreesen R (2006). Tumor-derived lactic acid modulates dendritic cell activation and antigen expression. Blood.

[305] Nasi A, Fekete T, Krishnamurthy A, Snowden S, Rajnavolgyi E, Catrina AI (2013). Dendritic cell reprogramming by endogenously produced lactic acid. J Immunol.

[306] Zhong H, Gutkin DW, Han B, Ma Y, Keskinov AA, Shurin MR (2014). Origin and pharmacological modulation of tumor-associated regulatory dendritic cells. Int J Cancer.

[307] Colegio OR, Chu NQ, Szabo AL, Chu T, Rhebergen AM, Jairam V (2014). Functional polarization of tumour-associated macrophages by tumour-derived lactic acid. Nature.

[308] Mitchem JB, Brennan DJ, Knolhoff BL, Belt BA, Zhu Y, Sanford DE (2013). Targeting tumor-infiltrating macrophages decreases tumor-initiating cells, relieves immunosuppression, and improves chemotherapeutic responses. Can Res.

[309] Lu CH, Yeh DW, Lai CY, Liu YL, Huang LR, Lee AY (2018). USP17 mediates macrophage-promoted inflammation and stemness in lung cancer cells by regulating TRAF2/TRAF3 complex formation. Oncogene.

[310] Deniz G, van de Veen W, Akdis M (2013). Natural killer cells in patients with allergic diseases. J Allergy Clin Immunol.

[311] Vivier E, Raulet DH, Moretta A, Caligiuri MA, Zitvogel L, Lanier LL (2011). Innate or adaptive immunity? The example of natural killer cells. Science.

[312] Luna JI, Grossenbacher SK, Murphy WJ, Canter RJ (2017). Targeting cancer stem cells with natural killer cell immunotherapy. Expert Opin Biol Ther.

[313] Di Tomaso T, Mazzoleni S, Wang E, Sovena G, Clavenna D, Franzin A (2010). Immunobiological characterization of cancer stem cells isolated from glioblastoma patients. Clin Cancer Res.

[314] Mittal V, El Rayes T, Narula N, McGraw TE, Altorki NK, Barcellos-Hoff MH (2016). The Microenvironment of Lung Cancer and Therapeutic Implications. Adv Exp Med Biol.

[315] Seif F, Torki Z, Zalpoor H, Habibi M, Pornour M (2023). Breast cancer tumor microenvironment affects Treg/IL-17-producing Treg/Th17 cell axis: Molecular and therapeutic perspectives. Mol Ther Oncolytics.

[316] Calcinotto A, Filipazzi P, Grioni M, Iero M, De Milito A, Ricupito A (2012). Modulation of microenvironment acidity reverses anergy in human and murine tumor-infiltrating T lymphocytes. Can Res.

[317] Bosticardo MAS, Losana G, Bernabei P, Forni G, Novelli F (2001). Biased activation of human T lymphocytes due to low extracellular pH is antagonized by B7/CD28 costimulation. Eur J Immunol.

[318] Roesch A, Vultur A, Bogeski I, Wang H, Zimmermann KM, Speicher D (2013). Overcoming intrinsic multidrug resistance in melanoma by blocking the mitochondrial respiratory chain of slow-cycling JARID1B(high) cells. Cancer Cell.

[319] Denise C, Paoli P, Calvani M, Taddei ML, Giannoni E, Kopetz S, Kazmi SMA, Pia MM, Pettazzoni P, Sacco E (2015). 5-fluorouracil resistant colon cancer cells are addicted to OXPHOS to survive and enhance stem-like traits. Oncotarget.

[320] Zhou Y, Zhou Y, Shingu T, Feng L, Chen Z, Ogasawara M (2011). Metabolic alterations in highly tumorigenic glioblastoma cells: preference for hypoxia and high dependency on glycolysis. J Biol Chem.

[321] Mao P, Joshi K, Li J, Kim SH, Li P, Santana-Santos L (2013). Mesenchymal glioma stem cells are maintained by activated glycolytic metabolism involving aldehyde dehydrogenase 1A3. Proc Natl Acad Sci USA.

[322] Barone A, Sengupta R, Warrington NM, Smith E, Wen PY, Brekken RA (2014). Combined VEGF and CXCR4 antagonism targets the GBM stem cell population and synergistically improves survival in an intracranial mouse model of glioblastoma. Oncotarget.

[323] Zhao J, Ma MZ, Ren H, Liu Z, Edelman MJ, Pan H (2013). Anti-HDGF targets cancer and cancer stromal stem cells resistant to chemotherapy. Clin Cancer Res.

[324] Cazet AS, Hui MN, Elsworth BL, Wu SZ, Roden D, Chan CL (2018). Targeting stromal remodeling and cancer stem cell plasticity overcomes chemoresistance in triple negative breast cancer. Nat Commun.

[325] Su S, Chen J, Yao H, Liu J, Yu S, Lao L (2018). CD10(+)GPR77(+)  Cancer-Associated Fibroblasts Promote Cancer Formation and Chemoresistance by Sustaining Cancer Stemness. Cell..

[326] Qian L, Tang Z, Yin S, Mo F, Yang X, Hou X (2018). Fusion of dendritic cells and cancer-associated fibroblasts for activation of anti-tumor cytotoxic t lymphocytes. J Biomed Nanotechnol.

[327] Kraman M, Bambrough PJ, Arnold JN, Roberts EW, Magiera L, Jones JO (2010). Suppression of antitumor immunity by stromal cells expressing fibroblast activation protein-alpha. Science.

[328] Loeffler M, Kruger JA, Niethammer AG, Reisfeld RA (2006). Targeting tumor-associated fibroblasts improves cancer chemotherapy by increasing intratumoral drug uptake. J Clin Investig.

[329] Kakarla S, Chow KK, Mata M, Shaffer DR, Song XT, Wu MF (2013). Antitumor effects of chimeric receptor engineered human T cells directed to tumor stroma. Mol Ther.

[330] Ferrer-Mayorga G, Gomez-Lopez G, Barbachano A, Fernandez-Barral A, Pena C, Pisano DG (2017). Vitamin D receptor expression and associated gene signature in tumour stromal fibroblasts predict clinical outcome in colorectal cancer. Gut.

[331] Herrera M, Herrera A, Dominguez G, Silva J, Garcia V, Garcia JM (2013). Cancer-associated fibroblast and M2 macrophage markers together predict outcome in colorectal cancer patients. Cancer Sci.

[332] Köksal H, Müller E, Inderberg EM, Bruland Ø, Wälchli S (2019). Treating osteosarcoma with CAR T cells. Scand J Immunol.

[333] Liu T, Han C, Wang S, Fang P, Ma Z, Xu L (2019). Cancer-associated fibroblasts: an emerging target of anti-cancer immunotherapy. J Hematol Oncol.

